# Three-Space as a Quantum Hyperlayer in 1+3 Dimensions: A Case Study in Quantum Space and Time

**DOI:** 10.3390/e27060549

**Published:** 2025-05-23

**Authors:** Marek Czachor

**Affiliations:** Instytut Fizyki i Informatyki Stosowanej, Politechnika Gdańska, 80-233 Gdańsk, Poland; mczachor@pg.edu.pl

**Keywords:** quantum time, quantum cosmology, hyperbolic space, invariant-time dynamics, relativistic harmonic oscillator

## Abstract

We discuss a formalism where a universe is identified with the support of a wave function propagating through space–time. The dynamics is of a squeezing type, with shrinking in time and expanding in space. As opposed to classical cosmology, the resulting universe is not a spacelike section of some space–time but a hyperlayer of a finite timelike width, a set which is not a three-dimensional submanifold of space–time. The universe is in superposition of different localizations in both space and time so that $x^{0}=ct$ has the same formal status of a position operator as the remaining three coordinates. We test the formalism on the example of a universe that contains a single harmonic oscillator, a generalization of the curvature-dependent Cariñena–Rañada–Santander (CRS) model. As opposed to the original CRS formulation, here, the curvature is not a parameter but a quantum observable, a function of the world-position operator. It is shown that asymptotically, for large values of the invariant evolution parameter τ, one reconstructs the standard quantum results, with one modification: The effective (renormalized) mass of the oscillator decreases with τ. The effect does not seem to be a peculiarity of harmonic oscillators, so one may speculate that masses of distant elementary quantum systems are greater than the values known from our quantum mechanical measurements.

## 1. Introduction

Our human brains have no difficulty imagining a two-dimensional surface, although “surfaces” known from physical experience are objects with non-zero thickness; hence, they are layers not surfaces. Realistic layers consist of atoms, but quantum mechanics describes atoms as objects that do not possess concrete positions in space. Rather, atoms are in superpositions of different localizations and thus are fundamentally delocalized. In effect, at the most fundamental level, we are always dealing with “quantum surfaces” that exist in superpositions of different geometries.

A similar situation is found in space–time physics. What we regard as “space” is modeled as a three-dimensional hypersurface of space–time, an object with zero thickness in timelike directions. Yet, our experience of time is fleeting and ephemeral. It is very difficult, if not impossible, to be truly here and now. In this sense, we do not have everyday experience with space as a three-dimensional submanifold of space–time. The experience of “now” seems as delocalized as the atoms that form a quantum surface. Perhaps what we regard as space is not a hypersurface but a hyperlayer.

This is the first intuition behind the present paper. The second intuition is related to the passage of time.

The passage of time means that even if we accept that “now” is somewhat uncertain, the past should eventually disappear and a sufficiently distant future should not yet exist. This type of behavior does not appear to have an equivalent in standard relativistic physics but is well known from quantum mechanics. Indeed, a propagating wave packet represents a particle in superposition of different localizations, concentrated around the point of maximal probability density. By the Ehrenfest theorem, the average position of the wave packet propagates along a solution of a classical Newton equation. A probability of finding the particle far behind, or far ahead of the wave packet is negligible. Now, it is enough to replace the space coordinate by $x^{0}=ct$ and treat the evolution parameter, τ say, as something distinct from $x^{0}$. A suitable wave packet propagates along $x^{0}$, and the result is precisely the dynamics where the past literally disappears, the future has not yet happened, and the uncertainty of “now” is represented by the width of the packet. A three-dimensional classical hypersurface is then obtained by an appropriate Ehrenfest theorem; in exactly the same way, a classical solution of Newton’s equation is found if we follow the average of the position operator.

Let us stress again that a hyperlayer in four dimensions should not be confused with a four-dimensional hypersurface of a (D>4)-dimensional space, similarly to an ordinary layer in three-dimensional space that is not a hypersurface in D>3 dimensions. In this sense, what we discuss should not be regarded as a version of a Pavšič-type quantized gravity [1,2], as the latter is based on higher-dimensional embeddings of four-dimensional hyper surfaces.

The idea we have just outlined is not entirely new. It seems that its first explicit formulation was given, for a 1+1 dimensional toy-model, in [3]. A generalization valid for any dimension was completed in [4] for the case of an empty universe. An inclusion of matter was briefly discussed in [4] as well, but a concrete study of a quantum mechanical system that exists and evolves in such a quantum space–time is still missing. The present paper is the first attempt of formulating and exactly solving a non-toy model in 1+3.

For obvious reasons, a harmonic oscillator is our first target. Standard non-relativistic oscillator is simple, well understood, exactly solvable by the factorization method, and is a cornerstone of field quantization. On the other hand, when it comes to relativistic physics, numerous possibilities occur. A generalization based on the Dirac equation was introduced by Moshinsky and Szczepaniak [5,6]. An alternative Dirac-type relativistic harmonic oscillator implicitly occurs in the so-called second Dirac equation [7], whose deep and exhaustive discussion can be found in [8]. A generalization where the kinetic term of the Hamiltonian is proportional to the d’Alembertian $\partial^{\mu }\partial_{\mu }$ in Minkowski space, while the potential is proportional to $x_{\mu }x^{\mu }$, is discussed in [9], generalizing the earlier works of Stueckelberg, Horwitz, and Piron [10,11,12]. The Horwitz Hamiltonian is also basically equivalent to Born’s “metric operator” $p_{\mu }p^{\mu }+x_{\mu }x^{\mu }$ (in dimensionless units), generating a spectrum of meson masses [13]. The formalism from [9] is similar to ours in that the evolution parameter is unrelated to $x^{0}$. In both formulations, the Hilbert spaces consist of functions integrable with respect to $d^{4}x$. However, both theories employ different Hamiltonians, different boundary conditions, and different correspondence principles with standard quantum mechanics. Our treatment of the harmonic oscillator is closest to the approach of Cariñena, Rañada, and Santander (CRS) [14,15], but the overall structure of our Schrödinger equation differs in the form of the free Hamiltonian, with empty universe as a reservoir for matter fields, a missing element of the formalism from [14,15].

We begin in Section 2 with a summary of the formalism proposed in [4]. We concentrate on the distinction between the universe and its background space–time and on the meaning of the boundary condition. In Section 3, we discuss in some detail the dynamics of the empty universe. An explicit example, adapted from [4], illustrates the evolution in 1+1 dimensional background (Appendix A discusses a similar but easier to interpret model, with the Minkowski space in (1+1)D replaced by the Euclidean plane). We also introduce the notion of a configuration-space universe. In Section 4, we give a concrete example of a single oscillator coupled to the universe whose background Minkowski space is 1+3 dimensional. We discuss both Schrödinger and interaction pictures. The interaction picture eliminates the free evolution of the universe for the price of making τ-dependent the Hamiltonian of the oscillator. This τ dependence will manifest itself in the form of the ground state, making the impression that the mass of the oscillator decreases with τ. The effective τ dependence of mass can be ignored at time scales available in typical quantum measurements but, in principle, could influence the interpretation of data from very distant objects. The next two sections discuss in detail the ground state of the oscillator. We first analyze in Section 5 an approximation that is simpler to analyze than the exact model from [14,15]. We concentrate on the ground state, but all the excited states can be found in Appendix B. We show that the ground state is a Gaussian but with respect to the geodesic position operator $\hat{r}=\hat{x}⊗\hat{\xi }$, whose eigenvalues represent the geodesic distance r=xξ computed along the hyperboloid $x^{2}=g_{\mu \nu }x^{\mu }x^{\nu }$. In Section 6, we perform an analogous analysis of the ground state for the CRS oscillator. As opposed to the original CRS formulation, the solution we find describes an oscillator in superposition of different and τ-dependent curvatures of the universe. The curvature that occurs in the solution is not, as opposed to [14,15], a parameter but a quantum observable, a fact proving that we indeed discuss a quantum oscillator in a quantum universe. It is also shown that differences between the exact CRS model and its much simpler approximate form are visible only for small τ, that is, in the early stages of evolution of our quantum universe. For τ corresponding to the Hubble time, both models are indistinguishable, a result useful from the point of view of the correspondence principle with standard quantum mechanics. Section 7 is devoted to the reduction 1+3→3, obtained by integrating out the width of the layer. We show that the resulting probability density is a bell-shaped curve similar to a Gaussian but more smeared out. Section 8 looks at the above issues from the perspective of a general two-body problem. In Appendix C, we discuss an alternative definition of the harmonic oscillator associated with a given Laplace–Beltrami operator. Unfortunately, the resulting potential does not lead to any known factorization of the Hamiltonian.

## 2. Expanding Quantum Universe

In the formalism proposed in [4] a universe is identified with a τ-dependent subset of the background Minkowski space M of signature (+−…−) in *D* dimensions. A point x∈M is said to belong to the universe if $\Psi_{\tau }\left(x\right)\neq 0$, where $\Psi_{\tau }$ is a solution of a certain Schrödinger-type equation,

(1) $$\begin{array}{c} i{\dot{\Psi }}_{\tau }=H\Psi_{\tau },\;\Psi_{\tau }=e^{-iH\tau }\Psi_{0}. \end{array}$$

The evolution parameter τ is dimensionless. $\Psi_{\tau }\left(x\right)$ is normalized in a τ-invariant way,

(2) $$\begin{array}{ccc} \langle \Psi_{\tau }|\Psi_{\tau }\rangle & = & \int_{V_{+}}d^{D}x{\left|\Psi_{\tau }\left(x\right)\right|}^{2}=1, \end{array}$$

but only for τ ≥ 0. For τ < 0, the norm can become τ dependent. Thus, (2) simultaneously defines a minimal possible value of τ and the corresponding arrow of time. Hamiltonian H is not self-adjoint, and yet it generates meaningful unitary dynamics of the universe. The non-self-adjointness is related to the existence of a minimal value of τ (see Appendix A for an analogous but more intuitive example formulated in the Euclidean plane $R^{2}$). For large τ, the asymptotic evolution of the universe should reconstruct the form of quantum mechanics we know from textbooks.

$V_{+}$ consists of future-timelike world-vectors $x^{\mu }$. The origin of the cone, x=0, is arbitrary. In standard classical Friedmann–Robertson–Walker (FRW)-type cosmology it might be natural to identify x=0 with the Big Bang, whereas in our model, the initial condition at τ=0 corresponds to a universe that is not localized at a point in space–time. The initial boundary condition reads $\Psi_{0}\left(x\right)=0$ if $x\notin V_{+}$. Such a $\Psi_{0}\left(x\right)$ vanishes outside of the cone $V_{+}$, including $\partial V_{+}$, the future light cone of the origin x=0. We additionally assume that the initial wavefunction $\Psi_{0}\left(x\right)$ is smooth and vanishes if $x=\sqrt{{\left(x^{0}\right)}^{2}-{\left(x^{1}\right)}^{2}-\ldots -{\left(x^{D-1}\right)}^{2}}$ does not belong to a certain open interval $]A_{0},B_{0}[\subset R_{+}$. The possible values of τ turn out to be related to the choice of $A_{0}$. Accordingly, different choices of $\Psi_{0}\left(x\right)$ imply different limitations on the minimal value of acceptable τ. All these properties would be impossible if H were self-adjoint, so that the non-self-adjointness of the Hamiltonian is an important conceptual ingredient of the theory.

The dynamics introduced in [4] guarantee that the support of $\Psi_{\tau }\left(x\right)$ involves only those *x* whose Minkowskian norm x belongs to $\left[A_{\tau },B_{\tau }\right]\subset R_{+}$, with $\lim_{\tau \to \infty }A_{\tau }=\infty$ and $\lim_{\tau \to \infty }\left(B_{\tau }-A_{\tau }\right)=0$ (the support is the closure of the set where a given function is nonzero, so the closed interval is not a typo). Unitarity of the evolution semigroup thus implies that the universe expands in space and shrinks in time. The unitary dynamics generated by H are of a squeezing type.

The usual spatial section of a FRW-type universe is here replaced by the support of $\Psi_{\tau }\left(x\right)$, but as τ increases, the timelike width $B_{\tau }-A_{\tau }$ shrinks to 0, so the support becomes asymptotically concentrated in a neighborhood of a hyperbolic section in $V_{+}$. In consequence, the universe is a subset of M that resembles a true material hyperbolic layer of a finite timelike width, propagating towards the future. We assume that at time scales of the order of the Hubble time, the timelike width of the layer is of the order of the Planck length, which leads to the estimate $\tau ∼10^{243}$ of the current value of τ, whereas one year is of the order of $\tau ∼10^{203}$ [4].

An analogous construction can be performed for spherical and flat universes.

## 3. Free Dynamics of an Empty Universe

The free Hamiltonian that describes an empty universe is given by

(3) $$\begin{array}{ccc} H_{0} & = & -\frac{\ell^{D}}{Dx^{D}}x^{\mu }\;i\partial_{\mu }. \end{array}$$

It generates a unitary semigroup. Recall that $x^{2}=g_{\mu \nu }x^{\mu }x^{\nu }$, where $g_{\mu \nu }$ is the Minkowski-space metric. Here, *ℓ* is a fundamental length parameter (the Planck length, say). Parametrizing the solution by means of x and the world-velocity $u^{\mu }=x^{\mu }/x$,

(4) $$\begin{array}{ccc} \psi_{\tau }\left(x,u\right) & = & \Psi_{\tau }\left(x\right), \end{array}$$

we find that $H_{0}$ is a generator of translations of the non-negative variable $x^{D}$,

(5) $$\begin{array}{ccc} H_{0}\psi_{\tau }\left(x,u\right) & = & -i\ell^{D}\frac{\partial }{\partial (x^{D})}\psi_{\tau }\left(x,u\right), \end{array}$$

which is not self-adjoint, an advantage in this context, as it turns out.

**Figure 1:**
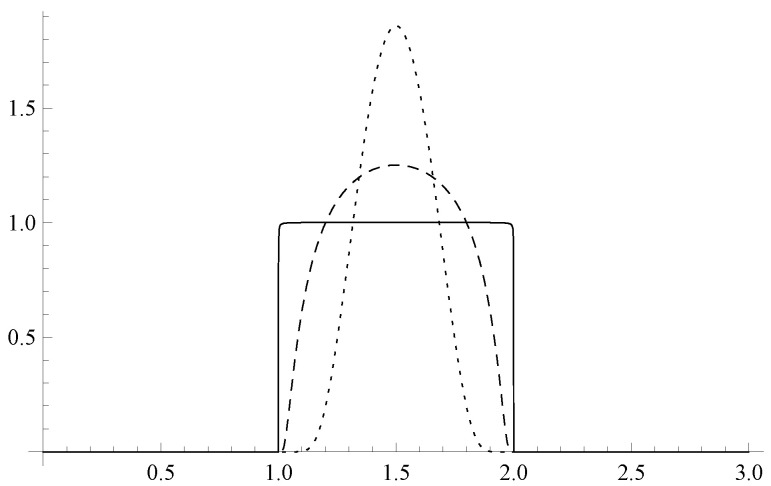
Normalized versions of (6) for $A_{0}=1$, $B_{0}=2$, and α=1 (dotted), α=10 (dashed), $\alpha =10^{4}$ (full). With increasing α, the function converges pointwise to the characteristic function of the interval $]A_{0},B_{0}[$. For finite α, the function is smooth, and all its derivatives vanish at both $A_{0}$ and $B_{0}$. The plotted functions are normalized with respect to $\langle f|f\rangle =\int_{0}^{\infty }dx\;{\left|f\left(x\right)\right|}^{2}$.

We assume that $\Psi_{0}\left(x\right)$ is smooth and compact-support in the variable x, vanishing outside of $]A_{0},B_{0}[\;\subset R_{+}$. An example of such a function is

(6) $$\begin{array}{c} \exp\frac{1}{\alpha \left(x-A_{0}\right)\left(x-B_{0}\right)},\;\mathrm{for}A_{0}<x<B_{0}, \end{array}$$

and 0 otherwise. Normalized versions of (6) for $A_{0}=1$, $B_{0}=2$, and various values of α are depicted in Figure 1. With α→∞, (6) converges pointwise to the characteristic function of $]A_{0},B_{0}[$. In many examples, we will tacitly assume that α is so large that (6) is practically indistinguishable from the characteristic function, and yet it remains smooth on the whole of $R_{+}$.

Under the above form of the initial condition, one finds, for $\ell^{D}\tau <x^{D}$,

(7) $$\begin{array}{ccc} \Psi_{\tau }\left(x\right) & = & \Psi_{0}\left({\left(\frac{x^{D}-\ell^{D}\tau }{x^{D}}\right)}^{1/D}x\right) \end{array}$$

(8) $$\begin{array}{ccc} & = & e^{-i\tau H_{0}}\Psi_{0}\left(x\right), \end{array}$$

whereas for $0\leq x^{D}\leq \ell^{D}\tau$, the solution vanishes identically,

(9) $$\begin{array}{ccc} \Psi_{\tau }\left(x\right) & \equiv & 0. \end{array}$$

The limiting value $x^{D}=\ell^{D}\tau$ defines the *gap hyperboloid*,

(10) $$\begin{array}{c} x^{2}={\left(x^{0}\right)}^{2}-{\left(x^{1}\right)}^{2}-\ldots -{\left(x^{D-1}\right)}^{2}=\ell^{2}\tau^{2/D}. \end{array}$$

**Figure 2:**
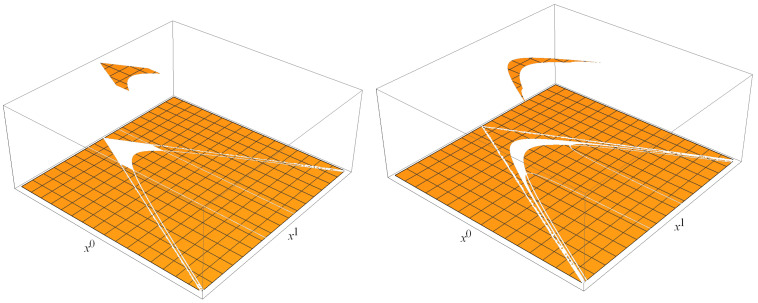
Space as an evolving quantum hyperlayer of space–time [4]. Plot of (7) and (8) with the initial condition (11) at τ=0 (left) and its evolved version for τ=1 (right) in a D=1+1 Minkowski space, in units where ℓ=1. $\Psi_{\tau }\left(x\right)$ at τ=1 is thinner and wider than $\Psi_{\tau }\left(x\right)$ at τ=0. A space–time gap occurs between the support of $\Psi_{1}\left(x\right)$ and the light cone. In 1+3 dimensions, the hyperboloid that determines the gap is given by $\ell^{2}\sqrt{\tau }=c^{2}t^{2}-x^{2}$, as contrasted with $c^{2}t^{2}-x^{2}∼\tau^{2}$, as one might expect on the basis of intuitions developed in special relativity. Note that the support of $\Psi_{\tau }\left(x\right)$ is not restricted to a single hyperbolic space. Rather, $\Psi_{\tau }\left(x\right)$ is a superposition of functions defined on hyperbolic spaces of different curvatures, corresponding to different values of $x^{2}=g_{\mu \nu }x^{\mu }x^{\nu }$. What we regard as the universe, is the set of those $x^{\mu }$ in Minkowski space where $\Psi_{\tau }\left(x\right)\neq 0$. The universe is not a (D−1)-dimensional submanifold of the Minkowski space but a *D*-dimensional open subset of the Minkowski space. This is the new paradigm. Harmonic oscillators discussed in the present paper involve less trivial forms of $\Psi_{\tau }\left(x\right)$.

An example of such a dynamic in D=1+1 is illustrated in Figure 2 (adapted from [4]), with the initial condition

(11) $$\begin{array}{c} \Psi_{0}\left(x\right)=\left\{\begin{array}{cc} 1 & \mathrm{for}|x^{1}|<1,\;{\left(x^{0}\right)}^{2}-{\left(x^{1}\right)}^{2}<1,\;x_{0}>0\\ 0 & \mathrm{otherwise} \end{array}\right. \end{array}$$

We assume that the jumps in (11) approximate a function of the form (6).

Before one fills the universe with matter, one needs an analogous formulation of configuration spaces. An *N*-point configuration-space empty-universe extension is defined by

(12) $$\begin{array}{ccc} \Psi_{\tau }\left(x_{1},\ldots ,x_{N}\right) & = & \Psi_{0}\left({\left(\frac{x_{1}^{D}-\ell^{D}\tau }{x_{1}^{D}}\right)}^{1/D}x_{1},\ldots ,{\left(\frac{x_{N}^{D}-\ell^{D}\tau }{x_{N}^{D}}\right)}^{1/D}x_{N},\right), \end{array}$$

which is equivalent to

(13) $$\begin{array}{ccc} \psi_{\tau }\left(x_{1},u_{1},\ldots ,x_{N},u_{N}\right) & = & \psi_{0}\left(\sqrt[{D}]{x_{1}^{D}-\ell^{D}\tau },u_{1},\ldots ,\sqrt[{D}]{x_{N}^{D}-\ell^{D}\tau },u_{N}\right) \end{array}$$

with the Hamiltonian

(14) $$\begin{array}{ccc} H_{0} & = & -\sum_{j=1}^{N}\frac{\ell^{D}}{Dx_{j}^{D}}x_{j}^{\mu }\;i\frac{\partial }{\partial x_{j}^{\mu }}=-i\ell^{D}\sum_{j=1}^{N}\frac{\partial }{\partial (x_{j}^{D})}, \end{array}$$

and the norm

(15) $$\begin{array}{ccc} \langle \Psi_{\tau }|\Psi_{\tau }\rangle & = & \int_{V_{+}}d^{D}x_{1}\ldots \int_{V_{+}}d^{D}x_{N}{\left|\Psi_{\tau }\left(x_{1},\ldots ,x_{N}\right)\right|}^{2} \end{array}$$

(16) $$\begin{array}{ccc} & = & \int_{0}^{\infty }dx_{1}\;x_{1}^{D-1}\int_{u_{1}^{2}=1}du_{1}\ldots \int_{0}^{\infty }dx_{N}\;x_{N}^{D-1}\int_{u_{N}^{2}=1}du_{N}\;{\left|\psi_{\tau }\left(x_{1},u_{1},\ldots ,x_{N},u_{N}\right)\right|}^{2}. \end{array}$$

Here, $du_{j}$ denotes an invariant measure on the world-velocity hyperboloid $u_{j}^{2}=g_{\mu \nu }u_{j}^{\mu }u_{j}^{\nu }=1$. The right-hand-side of (14) is applicable to functions that involve the world-velocity parametrization of the form (13). This point is somewhat tricky, as illustrated in Appendix B by Hamiltonians (A49) and (A50).

Now that we know how to describe the dynamics of an empty universe, it remains to add matter. We have two goals in mind. First of all, we have to test on some well-understood quantum mechanical models the asymptotic correspondence principle. The latter means that for very large τ, the theory should look like ordinary quantum mechanics, where the integrals are over $R^{D-1}$ and not $R^{D}$. Secondly, we should understand if and how the presence of matter influences a geometry of the universe, that is, the probability density $|\Psi_{\tau }\left(x_{1},\ldots ,x_{N}\right){|}^{2}$. A nontrivial modification of the Hamiltonian,

(17) $$\begin{array}{c} H=H_{0}\mapsto H_{0}+H_{1}, \end{array}$$

can, in principle, influence the set of points that satisfy $|\Psi_{\tau }\left(x_{1},\ldots ,x_{N}\right){|}^{2}\neq 0$. As the universe is the set of those *x* that have non-zero probability density, the same remark applies to the configuration-space universe.

An inclusion of matter can be accompanied by modifications of background space–time geometry. As an illustration of the possibility, consider the approach to quantum gravity based on precanonical field quantization [16,17]. Here, one begins with a Dirac-type equation for a Clifford-algebra-valued wave function f(ω,x), where ω stands for a tetrad-formalism connection defined at the space–time point *x*. The four space–time coordinates are here parameters, similarly to time *t* in non-relativistic quantum mechanics (or our invariant parameter τ), whereas the components of the connection play the role of configuration space coordinates. The precanonical scalar product involves integration over the 24 real components of ω but not over the four $x^{\mu }$,

(18) $$\begin{array}{c} \langle f\left(x\right)|g\left(x\right)\rangle =\int d^{24}\omega \;\mathrm{Tr}\left(\bar{f(\omega ,x)}g\left(\omega ,x\right)\right). \end{array}$$

Now, assume that f(ω,x) is a state of the quantized gravitational field at a space–time point *x*, whereas $\Psi_{0}\left(x\right)$ is smooth, compact-support in the variable $x=\sqrt{x_{\mu }x^{\mu }}$ and vanishes outside of some $]A_{0},B_{0}[\;\subset R_{+}$. With the initial condition $\Psi_{0}\left(\omega ,x\right)=\Psi_{0}\left(x\right)f\left(\omega ,x\right)$, we obtain the state of the universe at invariant cosmic time τ,

(19) $$\begin{array}{c} \Psi_{\tau }\left(\omega ,x\right)=e^{-iH\tau }\Psi_{0}\left(\omega ,x\right), \end{array}$$

with H given by (3). The norm of the full state will be then given by

(20) $$\begin{array}{ccc} \langle \Psi_{\tau }|\Psi_{\tau }\rangle & = & \langle \Psi_{0}|\Psi_{0}\rangle \end{array}$$

(21) $$\begin{array}{ccc} & = & \int d^{D}x{\left|\Psi_{0}\left(x\right)\right|}^{2}\int d^{24}\omega \;\mathrm{Tr}\left(\bar{f(\omega ,x)}f\left(\omega ,x\right)\right). \end{array}$$

However, in the present paper, we consider a simplified problem, with the universe whose background space–time is Minkowskian (hence with the trivial vanishing connection ω=0) but contains a single harmonic oscillator.

## 4. Quantum Oscillator in D=1+3

We skip the intermediate stage of a general two-body problem (postponed till Section 8) and directly concentrate on the configuration space of a relative coordinate. To this end, let us parametrize a future-pointing timelike world vector in (1+3)-dimensional configuration Minkowski space by the “polar relative coordinates”,

(22) $$\begin{array}{ccc} x^{0} & = & ct=x\cosh\xi ,\;x>0,\;\xi \geq 0, \end{array}$$

(23) $$\begin{array}{ccc} x^{1} & = & x\sinh\xi \sin\theta \cos\varphi ,\;0\leq \theta \leq \pi ,\;0\leq \varphi <2\pi , \end{array}$$

(24) $$\begin{array}{ccc} x^{2} & = & x\sinh\xi \sin\theta \sin\varphi , \end{array}$$

(25) $$\begin{array}{ccc} x^{3} & = & x\sinh\xi \cos\theta . \end{array}$$

In D=1+3, the Hamiltonian consists of the empty-universe free part, $H_{0}$,

(26) $$\begin{array}{ccc} H_{0}\psi_{\tau }\left(x,\xi ,\theta ,\varphi \right) & = & -i\ell^{4}\frac{\partial }{\partial (x^{4})}\psi_{\tau }\left(x,\xi ,\theta ,\varphi \right), \end{array}$$

and the matter-field interaction part,

(27) $$\begin{array}{ccc} H_{1} & = & \epsilon H, \end{array}$$

where *H* is some “ordinary” Hamiltonian that describes a quantum system on the hyperboloid $x^{2}=g_{\mu \nu }x^{\mu }x^{\nu }>0$. ϵ is a parameter that makes $H_{1}$ dimensionless like τ. We shall concentrate on *H* describing some form of a relativistic harmonic oscillator.

The Minkowski-space metric satisfies

(28) $$\begin{array}{ccc} g_{\mu \nu }dx^{\mu }dx^{\nu } & = & {\left(dx^{0}\right)}^{2}-{\left(dx^{1}\right)}^{2}-{\left(dx^{2}\right)}^{2}-{\left(dx^{3}\right)}^{2} \end{array}$$

(29) $$\begin{array}{ccc} & = & dx^{2}-x^{2}d\xi^{2}-x^{2}\sinh^{2}\xi \;d\theta^{2}-x^{2}\sinh^{2}\xi \sin^{2}\theta \;d\varphi^{2}. \end{array}$$

The corresponding Jacobian,

(30) $$\begin{array}{ccc} d^{4}x & = & dx^{0}dx^{1}dx^{2}dx^{3} \end{array}$$

(31) $$\begin{array}{ccc} & = & dx\;d\xi \;d\theta \;d\varphi \;x^{3}\sinh^{2}\xi \sin\theta , \end{array}$$

is consistent with Hermiticity of the polar-form d’Alembertian,

(32) $$\begin{array}{ccc} □ & = & \frac{\partial^{2}}{\partial {\left(x^{0}\right)}^{2}}-\frac{\partial^{2}}{\partial {\left(x^{1}\right)}^{2}}-\frac{\partial^{2}}{\partial {\left(x^{2}\right)}^{2}}-\frac{\partial^{2}}{\partial {\left(x^{3}\right)}^{2}} \end{array}$$

(33) $$\begin{array}{ccc} & = & \frac{1}{x^{3}}\frac{\partial }{\partial x}x^{3}\frac{\partial }{\partial x}-\frac{1}{x^{2}\sinh^{2}\xi }\frac{\partial }{\partial \xi }\sinh^{2}\xi \frac{\partial }{\partial \xi }-\frac{1}{x^{2}\sinh^{2}\xi }\left(\frac{1}{\sin\theta }\frac{\partial }{\partial \theta }\sin\theta \frac{\partial }{\partial \theta }+\frac{1}{\sin^{2}\theta }\frac{\partial^{2}}{\partial \varphi^{2}}\right), \end{array}$$

if appropriate boundary conditions are imposed. The operator,

(34) $$\begin{array}{ccc} \Delta_{x} & = & \frac{1}{x^{2}\sinh^{2}\xi }\frac{\partial }{\partial \xi }\sinh^{2}\xi \frac{\partial }{\partial \xi }+\frac{1}{x^{2}\sinh^{2}\xi }\left(\frac{1}{\sin\theta }\frac{\partial }{\partial \theta }\sin\theta \frac{\partial }{\partial \theta }+\frac{1}{\sin^{2}\theta }\frac{\partial^{2}}{\partial \varphi^{2}}\right), \end{array}$$

is the Laplace–Beltrami operator on the hyperboloid $x^{2}=g_{\mu \nu }x^{\mu }x^{\nu }>0$.

There are numerous ways of defining a harmonic oscillator in hyperbolic geometry (cf. [6,9] and Appendix A), but we find it simplest to consider a potential proportional to $\tanh^{2}\xi$ [14,15],

(35) $$\begin{array}{ccc} H & = & -\frac{\hbar^{2}}{2\mu }\Delta_{x}+\frac{\mu \omega^{2}x^{2}\tanh^{2}\xi }{2}. \end{array}$$

A world-vector (ct,x) satisfies in polar coordinates ${\left|x\right|}^{2}=x^{2}\sinh^{2}\xi$, so the potential

(36) $$\begin{array}{ccc} \frac{\mu \omega^{2}x^{2}\sinh^{2}\xi }{2\cosh^{2}\xi } & = & \frac{\mu \omega^{2}{\left|x\right|}^{2}}{2}\frac{1}{1+\sinh^{2}\xi }=\frac{\mu \omega^{2}{\left|x\right|}^{2}}{2}\frac{1}{1+{\left|x\right|}^{2}/x^{2}}\approx \frac{\mu \omega^{2}{\left|x\right|}^{2}}{2}, \end{array}$$

reconstructs the usual harmonic oscillator if |x| is of the size available in present-day experiments, while x is of the order of the Hubble radius of the universe. This type of approximation agrees with the one for the measure on the hyperboloid,

(37) $$\begin{array}{c} \frac{d^{3}x}{\sqrt{1+{\left|x\right|}^{2}/x^{2}}}\approx d^{3}x, \end{array}$$

which characterizes the correspondence principle with standard quantum mechanics.

For small values of ξ, the potential takes another interesting form, namely,

(38) $$\begin{array}{c} \frac{\mu \omega^{2}x^{2}\tanh^{2}\xi }{2}\approx \frac{\mu \omega^{2}x^{2}\xi^{2}}{2}=\frac{\mu \omega^{2}r^{2}}{2}, \end{array}$$

where r=xξ is the geodesic distance computed along the hyperboloid. Actually, the right-hand side of (38) is a natural alternative definition of the potential if one assumes that the physical distance between interacting objects should be given in terms of the geodesic distance r and not in terms of |x|, as the latter is not a geometrically intrinsic characteristic of the hyperboloid.

Perhaps we can obtain a more illuminating picture of the potential by writing it as follows:

(39) $$\begin{array}{ccc} \frac{\mu \omega^{2}x^{2}\sinh^{2}\xi }{2\cosh^{2}\xi } & = & \frac{\mu \omega^{2}x^{2}}{2}\frac{{\left(x^{1}\right)}^{2}+{\left(x^{2}\right)}^{2}+{\left(x^{3}\right)}^{2}}{{\left(x^{0}\right)}^{2}}, \end{array}$$

with ${\left(x^{0}\right)}^{2}>{\left(x^{1}\right)}^{2}+{\left(x^{2}\right)}^{2}+{\left(x^{3}\right)}^{2}$, showing that the possible three-space position of the oscillator is limited by the light cone x=0, the boundary of the background space–time.

The full Schrödinger equation,

(40) $$\begin{array}{ccc} i{\dot{\psi }}_{\tau }\left(x,\xi ,\theta ,\varphi \right) & = & -i\ell^{4}\frac{\partial }{\partial (x^{4})}\psi_{\tau }\left(x,\xi ,\theta ,\varphi \right)+\epsilon \left(-\frac{\hbar^{2}}{2\mu }\Delta_{x}+\frac{\mu \omega^{2}x^{2}\tanh^{2}\xi }{2}\right)\psi_{\tau }\left(x,\xi ,\theta ,\varphi \right), \end{array}$$

can be partly separated by means of

(41) $$\begin{array}{ccc} \psi_{lm,\tau }\left(x,\xi ,\theta ,\varphi \right) & = & \phi_{l,\tau }\left(x,\xi \right)Y_{lm}\left(\theta ,\varphi \right), \end{array}$$

(42) $$\begin{array}{ccc} i{\dot{\phi }}_{l,\tau }\left(x,\xi \right) & = & -i\ell^{4}\frac{\partial }{\partial (x^{4})}\phi_{l,\tau }\left(x,\xi \right)\\ & \;\;\; & +\epsilon \left(-\frac{\hbar^{2}}{2\mu }\frac{1}{x^{2}\sinh^{2}\xi }\frac{\partial }{\partial \xi }\sinh^{2}\xi \frac{\partial }{\partial \xi }+\frac{\hbar^{2}}{2\mu }\frac{l(l+1)}{x^{2}\sinh^{2}\xi }+\frac{\mu \omega^{2}x^{2}\tanh^{2}\xi }{2}\right)\phi_{l,\tau }\left(x,\xi \right), \end{array}$$

because the angular momentum operator,

(43) $$\begin{array}{c} J^{2}=-\hbar^{2}\left(\frac{1}{\sin\theta }\frac{\partial }{\partial \theta }\sin\theta \frac{\partial }{\partial \theta }+\frac{1}{\sin^{2}\theta }\frac{\partial^{2}}{\partial \varphi^{2}}\right), \end{array}$$

commutes with the total Hamiltonian, $H=H_{0}+H_{1}$.

The free Hamiltonian, $H_{0}$, is a generator of translations of the non-negative variable $x^{4}$. It effectively replaces x by $x\left(\tau \right)=\sqrt[{4}]{x^{4}+\ell^{4}\tau }$. The next step is therefore the transition to the interaction picture,

(44) $$\begin{array}{ccc} \Phi_{l,\tau }\left(x,\xi \right) & = & e^{\tau \ell^{4}\frac{\partial }{\partial (x^{4})}}\phi_{l,\tau }\left(x,\xi \right)=\phi_{l,\tau }\left(x\left(\tau \right),\xi \right). \end{array}$$

The equation to solve,

(45) $$\begin{array}{ccc} i{\dot{\Phi }}_{l,\tau }\left(x,\xi \right) & = & \epsilon \left(-\frac{\hbar^{2}}{2\mu }\frac{1}{x{\left(\tau \right)}^{2}\sinh^{2}\xi }\frac{\partial }{\partial \xi }\sinh^{2}\xi \frac{\partial }{\partial \xi }+\frac{\hbar^{2}}{2\mu }\frac{l(l+1)}{x{\left(\tau \right)}^{2}\sinh^{2}\xi }+\frac{\mu \omega^{2}x{\left(\tau \right)}^{2}\tanh^{2}\xi }{2}\right)\Phi_{l,\tau }\left(x,\xi \right), \end{array}$$

is equivalent to a harmonic oscillator on a space of constant but τ-dependent negative curvature. Recall that the solution is normalized by means of

(46) $$\begin{array}{ccc} \langle \Phi |\Phi \rangle & = & \int_{0}^{\infty }dx\;x^{3}\int_{0}^{\infty }d\xi \;\sinh^{2}\xi \;{\left|\Phi \left(x,\xi \right)\right|}^{2}. \end{array}$$

Notice that not only is the curvature τ-dependent, but it is not a classical parameter, as opposed to the standard literature of the subject. This is a quantum observable, as quantum as the position operator, since one integrates over x in (46). This universe is truly quantum and dynamic. It exists in the superposition of different curvatures.

## 5. Interlude: Ground State for Small ξ

Although Schrödinger Equation (45) can be solved exactly, let us first concentrate on the approximation valid for small ξ, as it will help us to develop physical intuitions concerning the nature of the solution. Setting sinhξ≈tanhξ≈ξ and l=0 (as we search for the ground state), we obtain

(47) $$\begin{array}{ccc} i{\dot{\Phi }}_{0,\tau }\left(x,\xi \right) & = & \epsilon \left(-\frac{\hbar^{2}}{2\mu }\frac{1}{x{\left(\tau \right)}^{2}\xi^{2}}\frac{\partial }{\partial \xi }\xi^{2}\frac{\partial }{\partial \xi }+\frac{\mu \omega^{2}x{\left(\tau \right)}^{2}\xi^{2}}{2}\right)\Phi_{0,\tau }\left(x,\xi \right), \end{array}$$

with the normalization

(48) $$\begin{array}{ccc} \langle \Phi |\Phi \rangle & = & \int_{0}^{\infty }dx\;x^{3}\int_{0}^{\infty }d\xi \;\xi^{2}\;{\left|\Phi \left(x,\xi \right)\right|}^{2}=1. \end{array}$$

The form (48) of the scalar product had to be modified in order to maintain the Hermiticity of the Laplacian in (47).

Note that (47) and (48) can be alternatively interpreted as an exact model in a spatially flat universe, where spacelike distances are computed by means of the hyperbolic geodesic distances. Such a flat universe is not equivalent to the Minkowski space and yet employs the Minkowski space as its background space-time—an interesting option to contemplate in some future work, especially in the context of the lambda cold dark matter (ΛCDM) cosmology.

Now, define $F_{\tau }\left(x,\xi \right)=\Phi_{0,\tau }\left(x,\xi \right)\xi$. Then

(49) $$\begin{array}{ccc} i{\dot{F}}_{\tau }\left(x,\xi \right) & = & \epsilon \left(-\frac{\hbar^{2}}{2\mu }\frac{1}{x{\left(\tau \right)}^{2}}\frac{\partial^{2}}{\partial \xi^{2}}+\frac{\mu \omega^{2}x{\left(\tau \right)}^{2}\xi^{2}}{2}\right)F_{\tau }\left(x,\xi \right) \end{array}$$

(50) $$\begin{array}{ccc} & = & \epsilon \hbar \omega \left(a{\left(x,\tau \right)}^{†}a\left(x,\tau \right)+\frac{1}{2}\right)F_{\tau }\left(x,\xi \right). \end{array}$$

The creation and annihilation operators,

(51) $$\begin{array}{ccc} a\left(x,\tau \right)F_{\tau }\left(x,\xi \right) & = & \frac{1}{\sqrt{\hbar \omega }}\left(\frac{\hbar }{\sqrt{2\mu }}\frac{1}{x(\tau )}\frac{\partial }{\partial \xi }+\sqrt{\frac{\mu }{2}}\omega x\left(\tau \right)\xi \right)F_{\tau }\left(x,\xi \right), \end{array}$$

(52) $$\begin{array}{ccc} a{\left(x,\tau \right)}^{†}F_{\tau }\left(x,\xi \right) & = & \frac{1}{\sqrt{\hbar \omega }}\left(-\frac{\hbar }{\sqrt{2\mu }}\frac{1}{x(\tau )}\frac{\partial }{\partial \xi }+\sqrt{\frac{\mu }{2}}\omega x\left(\tau \right)\xi \right)F_{\tau }\left(x,\xi \right), \end{array}$$

satisfy the usual algebra,

(53) $$\begin{array}{ccc} \left[a\left(\hat{x},\tau \right),a{\left(\hat{x},\tau \right)}^{†}\right] & = & I. \end{array}$$

The hat in $\hat{x}$ reminds us that x is an eigenvalue of $\hat{x}$. The ground state satisfies $a\left(x,\tau \right)F_{0,\tau }\left(x,\xi \right)=0$,

(54) $$\begin{array}{ccc} \frac{\partial F_{0,\tau }\left(x,\xi \right)}{\partial \xi } & = & -\frac{\mu \omega }{\hbar }\xi \sqrt{x^{4}+\ell^{4}\tau }F_{0,\tau }\left(x,\xi \right), \end{array}$$

(55) $$\begin{array}{ccc} i{\dot{F}}_{0,\tau }\left(x,\xi \right) & = & \frac{\epsilon \hbar \omega }{2}F_{0,\tau }\left(x,\xi \right), \end{array}$$

and thus

(56) $$\begin{array}{ccc} F_{0,\tau }\left(x,\xi \right) & = & F_{0,\tau }\left(x,0\right)e^{-\frac{\mu \omega }{2\hbar }\xi^{2}\sqrt{x^{4}+\ell^{4}\tau }}, \end{array}$$

(57) $$\begin{array}{ccc} F_{0,\tau }\left(x,\xi \right) & = & e^{-i\frac{\omega }{2}\epsilon \hbar \tau }F_{0,0}\left(x,\xi \right) \end{array}$$

(58) $$\begin{array}{ccc} & = & e^{-i\frac{\omega }{2}\epsilon \hbar \tau }F_{0,0}\left(x,0\right)e^{-\frac{\mu \omega }{2\hbar }x^{2}\xi^{2}}, \end{array}$$

or, equivalently,

(59) $$\begin{array}{ccc} \Phi_{0,\tau }\left(x,\xi \right) & = & e^{-i\frac{\omega }{2}\epsilon \hbar \tau }F_{0,0}\left(x,0\right)\xi^{-1}e^{-\frac{\mu \omega }{2\hbar }x^{2}\xi^{2}} \end{array}$$

(60) $$\begin{array}{ccc} & = & \phi_{0,\tau }\left(\sqrt[{4}]{x^{4}+\ell^{4}\tau },\xi \right). \end{array}$$

Returning to the Schrödinger picture, we finally find

(61) $$\begin{array}{ccc} \phi_{0,\tau }\left(x,\xi \right) & = & e^{-i\frac{\omega }{2}\epsilon \hbar \tau }F_{0,0}\left(\sqrt[{4}]{x^{4}-\ell^{4}\tau },0\right)\xi^{-1}e^{-\frac{\mu \omega }{2\hbar }\sqrt{x^{4}-\ell^{4}\tau }\xi^{2}}. \end{array}$$

Let us recall that $\phi_{0,0}\left(x,\xi \right)$ is non-zero only if $x\in \;]A_{0},B_{0}[\;\subset R_{+}$, for some $A_{0}$ and $B_{0}$, a fact implying that

(62) $$\begin{array}{ccc} \langle \phi_{0,\tau }|\phi_{0,\tau }\rangle & = & \int_{0}^{\infty }dx\;x^{3}\int_{0}^{\infty }d\xi \;\xi^{2}\;{\left|\phi_{0,\tau }\left(x,\xi \right)\right|}^{2} \end{array}$$

(63) $$\begin{array}{ccc} & = & \frac{1}{4}\int_{A_{0}^{4}+\ell^{4}\tau }^{B_{0}^{4}+\ell^{4}\tau }d\left(x^{4}\right)\int_{0}^{\infty }d\xi \;{\left|F_{0,0}\left(\sqrt[{4}]{x^{4}-\ell^{4}\tau },0\right)\right|}^{2}e^{-\frac{\mu \omega }{\hbar }\sqrt{x^{4}-\ell^{4}\tau }\xi^{2}} \end{array}$$

(64) $$\begin{array}{ccc} & = & \int_{A_{0}}^{B_{0}}dx\;x^{3}{\left|F_{0,0}\left(x,0\right)\right|}^{2}\int_{0}^{\infty }d\xi \;e^{-\frac{\mu \omega }{\hbar }x^{2}\xi^{2}} \end{array}$$

(65) $$\begin{array}{ccc} & = & \frac{1}{2}\sqrt{\frac{\pi \hbar }{\mu \omega }}\int_{A_{0}}^{B_{0}}dx\;x^{2}{\left|F_{0,0}\left(x,0\right)\right|}^{2}=1. \end{array}$$

In order to simplify the discussion, assume that $F_{0,0}\left(x,0\right)$ is given by a function proportional to (6) with a large value of α, say $\alpha =10^{100}$, so that $F_{0,0}\left(x,0\right)$, being smooth, is practically indistinguishable from the multiple $C\chi_{]A_{0},B_{0}[}\left(x\right)$ of the characteristic function $\chi_{]A_{0},B_{0}[}$ of the open interval $]A_{0},B_{0}[$. The normalization now reads

(66) $$\begin{array}{ccc} 1 & \approx & \frac{{\left|C\right|}^{2}}{6}\sqrt{\frac{\pi \hbar }{\mu \omega }}\left(B_{0}^{3}-A_{0}^{3}\right) \end{array}$$

so that

(67) $$\begin{array}{ccc} \phi_{0,\tau }\left(x,\xi \right) & \approx & \sqrt{\frac{6}{B_{0}^{3}-A_{0}^{3}}}{\left(\frac{\mu \omega }{\pi \hbar }\right)}^{1/4}e^{-i\frac{\omega }{2}\epsilon \hbar \tau }\chi_{]A_{0},B_{0}[}\left(\sqrt[{4}]{x^{4}-\ell^{4}\tau }\right)\xi^{-1}e^{-\frac{\mu \omega }{2\hbar }x^{2}\xi^{2}\sqrt{1-\frac{\ell^{4}\tau }{x^{4}}}}. \end{array}$$

The universe consists here of those events whose probability density is non-zero, $|\phi_{0,\tau }{\left(x,\xi \right)|}^{2}\neq 0$. Therefore, when analyzing the differences between (67) and the standard quantum prediction for the ground state, we can skip the characteristic function, still keeping in mind that its argument satisfies $A_{0}<\sqrt[{4}]{x^{4}-\ell^{4}\tau }<B_{0}$, with $A_{0}$ and $B_{0}$ determined by the initial condition for the universe at τ=0, hence, some 13 billion years ago. Moreover, it is clear that the role of non-relativistic time t is played here by ϵℏτ, while the product $x^{2}\xi^{2}=r^{2}$ is the square of the hyperboloid’s geodesic distance. The characteristic function implies that

(68) $$\begin{array}{c} 0<\frac{A_{0}^{2}}{x^{2}}<\sqrt{1-\frac{\ell^{4}\tau }{x^{4}}}<\frac{B_{0}^{2}}{x^{2}}<\frac{B_{0}^{2}}{\ell^{2}\sqrt{\tau }}, \end{array}$$

so $\phi_{0,\tau }\left(x,\xi \right)$ spreads along spacelike directions in a future neighborhood of the gap hyperboloid $x^{2}=\ell^{2}\sqrt{\tau }$, simultaneously shrinking in the timelike direction in a way determined by (68). All these properties are consistent with the analysis given in [4].

A qualitatively new element is given by the square root occurring in the Gaussian,

(69) $$\begin{array}{c} -\frac{\mu \omega }{2\hbar }r^{2}\sqrt{1-\frac{\ell^{4}\tau }{x^{4}}}, \end{array}$$

because, as a consequence of (68), we effectively find

(70) $$\begin{array}{c} \lim_{\tau \to \infty }\frac{\mu \omega }{\hbar }\sqrt{1-\frac{\ell^{4}\tau }{x^{4}}}=0. \end{array}$$

Assuming that *ℏ* is a fundamental constant, and taking into account that ω occurs in the oscillating term $e^{-i\frac{\omega }{2}\epsilon \hbar \tau }=e^{-i\frac{\omega }{2}t}$ in exactly the same way as the one we know from textbook quantum mechanics, we conclude that the Gaussian behavior of the geodesic variable r=xξ is controlled by the mass term

(71) $$\begin{array}{c} \mu \sqrt{1-\frac{\ell^{4}\tau }{x^{4}}}, \end{array}$$

which, accordingly, should be observed as decreasing with time. Obviously, in time scales available in present-day quantum measurements, we can assume that

(72) $$\begin{array}{c} \mu \sqrt{1-\frac{\ell^{4}\left(\tau +\delta \tau \right)}{x^{4}}}\approx \mu \sqrt{1-\frac{\ell^{4}\tau }{x^{4}}}, \end{array}$$

and thus, quantum oscillators are expected to behave as if their masses were time invariant. However, if what we observe is indeed the geodesic position r, then very distant objects should behave as if their masses were greater from the ones we know from our human laboratory measurements. Our conclusion is reminiscent of some results on time dependent masses of quantized scalar fields in both classical [18,19] and quantum cosmology [20,21,22].

All we have written above applies to the geodesic observable $\hat{r}=\hat{x}⊗\hat{\xi }$, whose eigenvalues are given by r=xξ. A measurement of $\hat{r}$ is therefore a measurement of a tensor product of two observables. One of them, namely, $\hat{x}$, determines location of the hyperboloid in the background Minkowski space (up to the uncertainty relation following from (68)). This is effectively a *measurement of quantum time*, as it approximately determines the value of τ. The measurement of $\hat{\xi }$ determines the position of the oscillator along the hyperboloid, so this is, essentially, a measurement of position. More precisely, the variable ξ has the status of a shape variable in Barbour’s sense (see [23] and the example discussed in [4]).

The two observables are distributed in space–time by means of the two reduced probability densities,

(73) $$\begin{array}{ccc} \rho_{\tau }\left(x\right) & = & \int_{0}^{\infty }d\xi \;x^{3}\xi^{2}\;{\left|\phi_{0,\tau }\left(x,\xi \right)\right|}^{2},\;\left(\mathrm{probability}\;\mathrm{density}\;\mathrm{of}\;\mathrm{quantum}\;\mathrm{time}\right), \end{array}$$

(74) $$\begin{array}{ccc} \rho_{\tau }\left(\xi \right) & = & \int_{0}^{\infty }dx\;x^{3}\xi^{2}\;{\left|\phi_{0,\tau }\left(x,\xi \right)\right|}^{2}=\rho_{0}\left(\xi \right),\;\left(\mathrm{probability}\;\mathrm{density}\;\mathrm{of}\;\mathrm{quantum}\;\mathrm{position}\right). \end{array}$$

Neither of them is the usual Gaussian, but the joint space–time probability distribution is Gaussian. $\rho_{\tau }\left(\xi \right)=\rho_{0}\left(\xi \right)$ in consequence of the same calculation as in the transition between (63) and (64).

## 6. The Exact Ground State

Let us consider the exact l=0 Equation (45) for $F_{\tau }\left(x,\xi \right)=\Phi_{0,\tau }\left(x,\xi \right)\sinh\xi$,

(75) $$\begin{array}{ccc} i{\dot{F}}_{\tau }\left(x,\xi \right) & = & \epsilon \frac{\hbar^{2}}{2\mu }\frac{1}{x{\left(\tau \right)}^{2}}\left(-\frac{\partial^{2}}{\partial \xi^{2}}+\frac{\mu^{2}\omega^{2}x{\left(\tau \right)}^{4}\tanh^{2}\xi }{\hbar^{2}}+1\right)F_{\tau }\left(x,\xi \right). \end{array}$$

The Hamiltonian in (75) can be factorized,

(76) $$\begin{array}{ccc} i{\dot{F}}_{\tau }\left(x,\xi \right) & = & \epsilon \frac{\hbar^{2}}{2\mu }\frac{1}{x{\left(\tau \right)}^{2}}\left(A{\left(x,\tau \right)}^{†}A\left(x,\tau \right)+\sqrt{\frac{1}{4}+\frac{\mu^{2}\omega^{2}x{\left(\tau \right)}^{4}}{\hbar^{2}}}+\frac{1}{2}\right)F_{\tau }\left(x,\xi \right), \end{array}$$

(77) $$\begin{array}{ccc} A(x,\tau ) & = & \frac{\partial }{\partial \xi }+\left(\sqrt{\frac{1}{4}+\frac{\mu^{2}\omega^{2}x{\left(\tau \right)}^{4}}{\hbar^{2}}}-\frac{1}{2}\right)\tanh\xi , \end{array}$$

(78) $$\begin{array}{ccc} A{\left(x,\tau \right)}^{†} & = & -\frac{\partial }{\partial \xi }+\left(\sqrt{\frac{1}{4}+\frac{\mu^{2}\omega^{2}x{\left(\tau \right)}^{4}}{\hbar^{2}}}-\frac{1}{2}\right)\tanh\xi . \end{array}$$

The ground state is annihilated by $A(\hat{x},\tau )$,

(79) $$\begin{array}{ccc} A\left(x,\tau \right)F_{0,\tau }\left(x,\xi \right), & = & 0, \end{array}$$

which implies

(80) $$\begin{array}{ccc} \frac{\partial F_{0,\tau }\left(x,\xi \right)}{\partial \xi } & = & -\left(\sqrt{\frac{1}{4}+\frac{\mu^{2}\omega^{2}x{\left(\tau \right)}^{4}}{\hbar^{2}}}-\frac{1}{2}\right)\tanh\xi F_{0,\tau }\left(x,\xi \right) \end{array}$$

and

(81) $$\begin{array}{ccc} \Phi_{0,\tau }\left(x,\xi \right) & = & \frac{F_{0,\tau }\left(x,\xi \right)}{\sinh\xi } \end{array}$$

(82) $$\begin{array}{ccc} & = & \frac{1}{\sinh\xi }{\left(\cosh\xi \right)}^{\frac{1}{2}-\sqrt{\frac{1}{4}+\frac{\mu^{2}\omega^{2}\left(x^{4}+\ell^{4}\tau \right)}{\hbar^{2}}}}F_{0,\tau }\left(x,0\right). \end{array}$$

Let us keep in mind that this is still the interaction-picture solution.

The τ-dependent equation,

(83) $$\begin{array}{ccc} i{\dot{F}}_{0,\tau }\left(x,\xi \right) & = & \epsilon \frac{\hbar^{2}}{2\mu }\frac{1}{x{\left(\tau \right)}^{2}}\left(\sqrt{\frac{1}{4}+\frac{\mu^{2}\omega^{2}x{\left(\tau \right)}^{4}}{\hbar^{2}}}+\frac{1}{2}\right)F_{0,\tau }\left(x,\xi \right), \end{array}$$

is solved by

(84) $$\begin{array}{ccc} F_{0,\tau }\left(x,\xi \right) & = & e^{-i\epsilon \frac{\hbar^{2}}{4\mu }\int_{0}^{\tau }d\tau^{\prime }\left(\sqrt{\frac{1}{x^{4}+\ell^{4}\tau^{\prime }}+\frac{4\mu^{2}\omega^{2}}{\hbar^{2}}}+\frac{1}{\sqrt{x^{4}+\ell^{4}\tau^{\prime }}}\right)}F_{0,0}\left(x,\xi \right), \end{array}$$

with $F_{0,0}\left(x,\xi \right)$ following from (82),

(85) $$\begin{array}{ccc} F_{0,0}\left(x,\xi \right) & = & {\left(\cosh\xi \right)}^{\frac{1}{2}-\sqrt{\frac{1}{4}+\frac{\mu^{2}\omega^{2}x^{4}}{\hbar^{2}}}}F_{0,0}\left(x,0\right). \end{array}$$

The integral

(86) $$\begin{array}{ccc} I(x,\omega ,\tau ) & = & \int_{0}^{\tau }d\tau^{\prime }\sqrt{\frac{1}{x^{4}+\ell^{4}\tau^{\prime }}+\frac{4\mu^{2}\omega^{2}}{\hbar^{2}}} \end{array}$$

is explicitly given by

(87) $$\begin{array}{ccc} I(x,\omega ,\tau ) & = & \frac{\hbar }{2\mu \omega }\frac{1}{\ell^{4}}\ln\left(\sqrt{1+\frac{4\mu^{2}\omega^{2}}{\hbar^{2}}\left(x^{4}+\ell^{4}\tau \right)}+\frac{2\mu \omega }{\hbar }\sqrt{x^{4}+\ell^{4}\tau }\right)+\frac{1}{\ell^{4}}\sqrt{1+\frac{4\mu^{2}\omega^{2}}{\hbar^{2}}\left(x^{4}+\ell^{4}\tau \right)}\sqrt{x^{4}+\ell^{4}\tau }\\ & \;\;\;= & -\frac{\hbar }{2\mu \omega }\frac{1}{\ell^{4}}\ln\left(\sqrt{1+\frac{4\mu^{2}\omega^{2}}{\hbar^{2}}x^{4}}+\frac{2\mu \omega }{\hbar }x^{2}\right)-\frac{1}{\ell^{4}}\sqrt{1+\frac{4\mu^{2}\omega^{2}}{\hbar^{2}}x^{4}}x^{2}. \end{array}$$

Note that

(88) $$\begin{array}{ccc} \lim_{\omega \to 0}\frac{\hbar }{2\mu \omega }\frac{1}{\ell^{4}}\ln\left(\sqrt{1+\frac{4\mu^{2}\omega^{2}}{\hbar^{2}}\left(x^{4}+\ell^{4}\tau \right)}+\frac{2\mu \omega }{\hbar }\sqrt{x^{4}+\ell^{4}\tau }\right) & = & \lim_{\omega \to 0}\frac{1}{\ell^{4}}\sqrt{1+\frac{4\mu^{2}\omega^{2}}{\hbar^{2}}\left(x^{4}+\ell^{4}\tau \right)}\sqrt{x^{4}+\ell^{4}\tau }\\ & = & \frac{\sqrt{x^{4}+\ell^{4}\tau }}{\ell^{4}}, \end{array}$$

hence,

(89) $$\begin{array}{ccc} I(x,0,\tau ) & = & \frac{2}{\ell^{4}}\left(\sqrt{x^{4}+\ell^{4}\tau }-x^{2}\right)=\int_{0}^{\tau }d\tau^{\prime }\frac{1}{\sqrt{x^{4}+\ell^{4}\tau^{\prime }}}, \end{array}$$

as implied by (87)–(88), can be cross-checked by direct integration. The full interaction-picture solution reads

(90) $$\begin{array}{ccc} \Phi_{0,\tau }\left(x,\xi \right) & = & e^{-i\epsilon \frac{\hbar^{2}}{4\mu }\left(I(x,\omega ,\tau )+I(x,0,\tau \right)}\frac{1}{\sinh\xi }{\left(\cosh\xi \right)}^{\frac{1}{2}-\sqrt{\frac{1}{4}+\frac{\mu^{2}\omega^{2}x^{4}}{\hbar^{2}}}}F_{0,0}\left(x,0\right), \end{array}$$

which translates in the Schrödinger picture into

(91) $$\begin{array}{ccc} \phi_{0,\tau }\left(x,\xi \right) & = & e^{-i\epsilon \frac{\hbar^{2}}{4\mu }\left(I\left(\sqrt[{4}]{x^{4}-\ell^{4}\tau },\omega ,\tau \right)+I\left(\sqrt[{4}]{x^{4}-\ell^{4}\tau },0,\tau \right)\right)}\frac{1}{\sinh\xi }{\left(\cosh\xi \right)}^{\frac{1}{2}-\frac{1}{2}\sqrt{1+\frac{4\mu^{2}\omega^{2}\left(x^{4}-\ell^{4}\tau \right)}{\hbar^{2}}}}F_{0,0}\left(\sqrt[{4}]{x^{4}-\ell^{4}\tau },0\right). \end{array}$$

For x>0, which we assume, the solution is normalized by

(92) $$\begin{array}{ccc} \langle \phi_{0,\tau }|\phi_{0,\tau }\rangle & = & \langle \phi_{0,0}|\phi_{0,0}\rangle =1 \end{array}$$

(93) $$\begin{array}{ccc} & = & \int_{0}^{\infty }dx\;x^{3}\int_{0}^{\infty }d\xi \;\sinh^{2}\xi \;{\left|\phi_{0,\tau }\left(x,\xi \right)\right|}^{2} \end{array}$$

(94) $$\begin{array}{ccc} & = & \int_{A_{0}}^{B_{0}}dx\;x^{3}\;{\left|F_{0,0}\left(x,0\right)\right|}^{2}\int_{0}^{\infty }d\xi \;{\left(\cosh\xi \right)}^{1-\sqrt{1+\frac{4\mu^{2}\omega^{2}x^{4}}{\hbar^{2}}}}, \end{array}$$

with

(95) ∫0∞dξ(coshξ)1−a=2a−1F1212(a+1),a;12(a+3);−1a+1+F1212(a−1),a;12(a+1);−1a−1,

for a>1. Equation (94) reconstructs the approximate result (64) in consequence of the limit

(96) $$\begin{array}{ccc} \lim_{x\to \infty }{\left(\cosh(r/x)\right)}^{1-\sqrt{1+\frac{4\mu^{2}\omega^{2}x^{4}}{\hbar^{2}}}} & = & e^{-\frac{\mu \omega }{\hbar }r^{2}}, \end{array}$$

and its uniform and fast convergence.

The phase factor $e^{-iS(\tau )}$ in (91) is given by

(97) $$\begin{array}{ccc} S(\tau ) & = & \epsilon \frac{\hbar^{2}}{4\mu }\left(I\left(\sqrt[{4}]{x^{4}-\ell^{4}\tau },\omega ,\tau \right)+I\left(\sqrt[{4}]{x^{4}-\ell^{4}\tau },0,\tau \right)\right). \end{array}$$

Its late-τ asymptotics should be compared with ωt/2=ωϵℏτ/2, occurring in (67).

To this end, we have to recall the support property of the initial condition at τ=0, and its consequence

(98) $$\begin{array}{c} A_{0}^{4}<x^{4}-\ell^{4}\tau <B_{0}^{4}, \end{array}$$

where $B_{0}-A_{0}$ is of the order of several light-minutes, roughly 1 astronomical unit AU (for a justification of the estimate, see [4]). Under these assumptions, we are interested in the asymptotic form of

(99) $$\begin{array}{ccc} I(\sqrt[{4}]{x^{4}-\ell^{4}\tau },\omega ,\tau ) & = & -\frac{\hbar }{2\mu \omega }\frac{1}{\ell^{4}}\ln\left(\sqrt{1+\frac{4\mu^{2}\omega^{2}}{\hbar^{2}}\left(x^{4}-\ell^{4}\tau \right)}+\frac{2\mu \omega }{\hbar }\sqrt{x^{4}-\ell^{4}\tau }\right)\\ & & -\frac{1}{\ell^{4}}\sqrt{1+\frac{4\mu^{2}\omega^{2}}{\hbar^{2}}\left(x^{4}-\ell^{4}\tau \right)}\sqrt{x^{4}-\ell^{4}\tau }\\ & & +\frac{\hbar }{2\mu \omega }\frac{1}{\ell^{4}}\ln\left(\sqrt{1+\frac{4\mu^{2}\omega^{2}}{\hbar^{2}}x^{4}}+\frac{2\mu \omega }{\hbar }x^{2}\right)+\frac{1}{\ell^{4}}\sqrt{1+\frac{4\mu^{2}\omega^{2}}{\hbar^{2}}x^{4}}x^{2}, \end{array}$$

(100) $$\begin{array}{ccc} I(\sqrt[{4}]{x^{4}-\ell^{4}\tau },0,\tau ) & = & -\frac{2}{\ell^{4}}\left(\sqrt{x^{4}-\ell^{4}\tau }-x^{2}\right), \end{array}$$

which, effectively, can be reduced by means of the gap-hyperboloid condition to $x\approx \ell \tau^{1/4}$,

$$\begin{array}{ccc} I\left(\sqrt[{4}]{x^{4}-\ell^{4}\tau },\omega ,\tau \right)+I\left(\sqrt[{4}]{x^{4}-\ell^{4}\tau },0,\tau \right) & \approx & \frac{\hbar }{2\mu \omega }\frac{1}{\ell^{4}}\ln\left(\sqrt{1+\frac{4\mu^{2}\omega^{2}}{\hbar^{2}}x^{4}}+\frac{2\mu \omega }{\hbar }x^{2}\right) \end{array}$$

(101) $$\begin{array}{ccc} & +\;\;\;= & +\frac{1}{\ell^{4}}\sqrt{1+\frac{4\mu^{2}\omega^{2}}{\hbar^{2}}x^{4}}x^{2}+\frac{2}{\ell^{4}}x^{2} \end{array}$$

(102) $$\begin{array}{ccc} & \approx & \frac{\hbar }{2\mu \omega }\frac{1}{\ell^{4}}\ln\left(\sqrt{\frac{4\mu^{2}\omega^{2}}{\hbar^{2}}x^{4}}+\frac{2\mu \omega }{\hbar }x^{2}\right)+\frac{1}{\ell^{4}}\sqrt{\frac{4\mu^{2}\omega^{2}}{\hbar^{2}}x^{4}}x^{2}+\frac{2}{\ell^{4}}x^{2} \end{array}$$

(103) $$\begin{array}{ccc} & \approx & \frac{\hbar }{2\mu \omega }\frac{1}{\ell^{4}}\ln\left(\frac{4\mu \omega }{\hbar }x^{2}\right)+\frac{1}{\ell^{4}}\frac{2\mu \omega }{\hbar }x^{4}+\frac{2}{\ell^{4}}x^{2} \end{array}$$

(104) $$\begin{array}{ccc} & \approx & \frac{1}{\ell^{4}}\frac{2\mu \omega }{\hbar }x^{4}\approx \frac{1}{\ell^{4}}\frac{2\mu \omega }{\hbar }\ell^{4}\tau . \end{array}$$

Asymptotically, for large τ, we obtain the expected result,

(105) $$\begin{array}{ccc} S(\tau ) & \approx & \epsilon \frac{\hbar^{2}}{4\mu }\frac{2\mu \omega }{\hbar }\tau =\frac{\omega }{2}\epsilon \hbar \tau =\frac{\omega }{2}t. \end{array}$$

## 7. 1+3→3 Reduction: Three-Space Probabilities

The three-space probability density is defined by either

(106) $$\begin{array}{ccc} \rho_{\tau }\left(\xi ,\theta ,\varphi \right) & = & \int dx\;x^{3}\sinh^{2}\xi \sin\theta {\left|\psi_{\tau }\left(x,\xi ,\theta ,\varphi \right)\right|}^{2}, \end{array}$$

or

(107) $$\begin{array}{ccc} \rho_{\tau }\left(\xi ,\theta ,\varphi \right) & = & \int dx\;x^{3}\xi^{2}\sin\theta {\left|\psi_{\tau }\left(x,\xi ,\theta ,\varphi \right)\right|}^{2}, \end{array}$$

if we work in the approximation sinhξ≈tanhξ≈ξ (or in a flat universe). In virtue of the initial condition, we assume the support of $\psi_{\tau }\left(x,\xi ,\theta ,\varphi \right)$ is restricted by the inequality

(108) $$\begin{array}{c} \sqrt[{4}]{A_{0}^{4}+\ell^{4}\tau }\leq x\leq \sqrt[{4}]{B_{0}^{4}+\ell^{4}\tau }, \end{array}$$

for some $0\leq A_{0}<B_{0}<\infty$. When we speak of the support, we mean, of course, the closure of the set of those $x^{\mu }$ where the wave function is non-zero. Hence, even for $A_{0}=0$ and τ=0, we may treat the argument $x^{\mu }$ of the wave function as a future-pointing timelike world vector, with x strictly positive.

A practical implication of inequality (108) is that asymptotically, for large τ, the solution is localized in a future neighborhood of the hyperboloid $x^{2}=\ell^{2}\sqrt{\tau }$, with τ counted out since the origin of the universe. This, on the other hand, implies that the present age of the universe, when referred to our human labs, is approximately equal to $\ell \sqrt[{4}]{\tau }$.

For large τ, the theory reconstructs standard quantum mechanics if we treat r=xξ as the measure of distance in position space. More precisely, r should be treated as the radial coordinate in spherical coordinates. However, the integration over x implies that r=xξ will not occur in the asymptotic three-space formulas. Therefore, in order to compare the three-space theory with standard 3D-space quantum mechanics, we have to introduce a parameter, *R*, representing an average x, averaged under the assumption of (108). Present-day quantum measurements may be expected to involve *R* of the order of 10–20 billion light-years. Accordingly, as another rule of thumb, we may assume that $r=R\xi \approx \xi \ell \sqrt[{4}]{\tau }$ is the radial coordinate known from quantum mechanics textbooks. At time scales δτ/τ≪1, available in our human galaxy-scale quantum measurements, we can assume $\xi \ell \sqrt[{4}]{\tau +\delta \tau }\approx \xi \ell \sqrt[{4}]{\tau }$. A variation of *r* with τ can be ignored as long as the asymptotic form of quantum mechanics is being used.

What we have just described is the correspondence principle with standard quantum mechanics. It is similar to the one introduced by Infeld and Schild [24] in their analysis of the Kepler problem. The difference is that [24] treats the hyperbolic space as the configuration space for 3-dimensional position-representation quantum mechanics, whereas in our formalism, the configuration space is Minkowskian (i.e., (1+3)-dimensional), and instead of a single hyperbolic geometry, the configuration space is a quantum superposition of different hyperbolic geometries (with different curvatures).

### 7.1. Approximate Three-Space Probabilities

We again begin with the approximation sinhξ≈tanhξ≈ξ. $F_{0,0}\left(x,0\right)$ is being given by a function of the form depicted in Figure 1, with large α, so that the differences with respect to the characteristic function of $]A_{0},B_{0}[$ can be ignored. For l=0, the dependence on spherical angles is trivial, so we are left with

(109) $$\begin{array}{ccc} \rho_{\tau }\left(\xi \right) & = & \int_{0}^{\infty }dx\;x^{3}\xi^{2}\;{\left|\phi_{0,\tau }\left(x,\xi \right)\right|}^{2} \end{array}$$

(110) $$\begin{array}{ccc} & = & \frac{3}{\left(B_{0}^{3}-A_{0}^{3}\right)\sqrt{\pi }}{\left(\frac{\hbar }{\mu \omega }\right)}^{3/2}\frac{e^{-\frac{\mu \omega }{\hbar }\xi^{2}A_{0}^{2}}\left(\frac{\mu \omega }{\hbar }\xi^{2}A_{0}^{2}+1\right)-e^{-\frac{\mu \omega }{\hbar }\xi^{2}B_{0}^{2}}\left(\frac{\mu \omega }{\hbar }\xi^{2}B_{0}^{2}+1\right)}{\xi^{4}}. \end{array}$$

In order to switch from the shape variable ξ to the asymptotic spherical coordinate r=Rξ (not to be confused with r=xξ), we employ the change of variables

(111) $$\begin{array}{ccc} \varrho_{0}\left(r\right) & = & R^{-1}\rho_{0}\left(R^{-1}r\right) \end{array}$$

(112) $$\begin{array}{ccc} & = & \frac{3R^{3}}{\left(B_{0}^{3}-A_{0}^{3}\right)\sqrt{\pi }}{\left(\frac{\hbar }{\mu \omega }\right)}^{3/2}\frac{e^{-\frac{A_{0}^{2}}{R^{2}}\frac{\mu \omega }{\hbar }r^{2}}\left(\frac{A_{0}^{2}}{R^{2}}\frac{\mu \omega }{\hbar }r^{2}+1\right)-e^{-\frac{B_{0}^{2}}{R^{2}}\frac{\mu \omega }{\hbar }r^{2}}\left(\frac{B_{0}^{2}}{R^{2}}\frac{\mu \omega }{\hbar }r^{2}+1\right)}{r^{4}}. \end{array}$$

**Figure 3:**
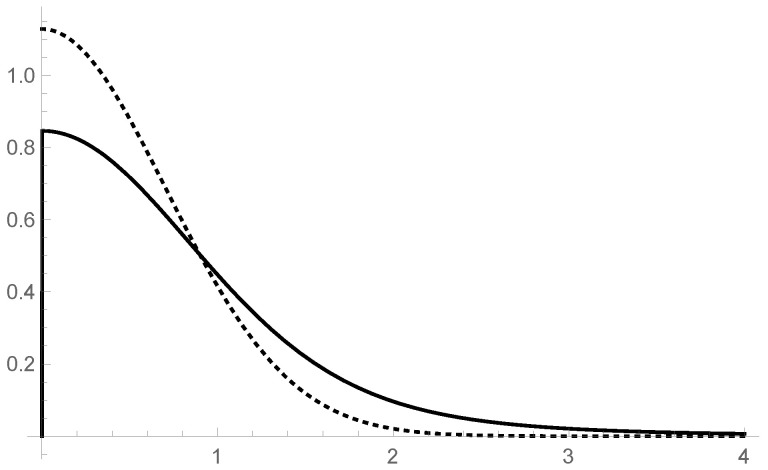
Gaussian (114) (dotted) versus $\varrho_{0}\left(r\right)$ (full) given by (113). The units are dimensionless, $\tilde{\mu }\omega /\hbar =1$, and the normalization is $\int_{0}^{\infty }dr\;\rho_{g}\left(r\right)=\int_{0}^{\infty }dr\;\varrho_{0}\left(r\right)=1$. The parameter that controls both densities, $\tilde{\mu }=\mu B_{0}^{2}/R^{2}$, effectively depends on τ, because asymptotically $R\approx \ell \sqrt[{4}]{\tau }$. The fact that wave functions are defined on space–time makes the reduced ground state additionally smeared out in the 3-position space. The effective τ dependence of $\tilde{\mu }$ can be ignored as long as the duration of quantum measurements, δτ, is negligible in comparison to the age τ of the universe, δτ/τ≪1.

To illustrate the form of $\varrho_{0}\left(r\right)$, let us take $A_{0}=0$ and denote by $\tilde{\mu }=\mu B_{0}^{2}/R^{2}$ the “renormalized mass”. The resulting density,

(113) $$\begin{array}{ccc} \varrho_{0}\left(r\right) & = & \frac{3}{\sqrt{\pi }}{\left(\frac{\hbar }{\tilde{\mu }\omega }\right)}^{3/2}\frac{1-e^{-\frac{\tilde{\mu }\omega }{\hbar }r^{2}}\left(\frac{\tilde{\mu }\omega }{\hbar }r^{2}+1\right)}{r^{4}}, \end{array}$$

is plotted in Figure 3, as compared to the Gaussian with the same parameters,

(114) $$\begin{array}{ccc} \rho_{g}\left(r\right) & = & 2\sqrt{\frac{\tilde{\mu }\omega }{\pi \hbar }}e^{-\frac{\tilde{\mu }\omega }{\hbar }r^{2}}. \end{array}$$

For a given τ, the universe is localized in a future neighborhood of the hyperboloid $x=\ell \sqrt[{4}]{\tau }$, so that for a negligible ratio |x|/x (typical of our-galaxy labs), the Minkowski-space time coordinate of quantum events, $x^{0}=ct$, is approximately equal to $\ell \sqrt[{4}]{\tau }$, a fact implying that

(115) $$\begin{array}{ccc} \tilde{\mu } & = & \mu \frac{B_{0}^{2}}{R^{2}}\approx \mu \frac{B_{0}^{2}}{\ell^{2}\sqrt{\tau }}\approx \mu \frac{B_{0}^{2}}{c^{2}t^{2}}\approx \mu \frac{B_{0}^{2}}{c^{2}{\left(t_{0}+\delta t\right)}^{2}}\approx \mu \frac{B_{0}^{2}}{c^{2}t_{0}^{2}}-2\mu \frac{B_{0}^{2}}{c^{2}t_{0}^{3}}\delta t=\mu_{0}\left(1-\frac{2\delta t}{t_{0}}\right), \end{array}$$

where $\mu_{0}$ and $t_{0}$ denote, respectively, the current value of mass of the oscillator and the current age of the universe. δt is the duration of the quantum measurement. Assuming $t_{0}$ is 10 billion years and δt a thousand years, we obtain

(116) $$\begin{array}{c} \tilde{\mu }\approx \mu_{0}-\delta \mu_{0}, \end{array}$$

with $\delta \mu_{0}/\mu_{0}∼10^{-7}$. The masses we are dealing with have decreased during the past millennium by some $10^{-5}$ percent.

Of course, one should not treat the above estimate too seriously—we are still at the level of an approximate toy model, with the universe “filled” with a single harmonic oscillator.

### 7.2. The Exact Three-Space Probabilities

Assuming that within the range of integration, $F_{0,0}\left(x,0\right)$ is well approximated by a constant *C*, we find

(117) $$\begin{array}{ccc} \rho_{\tau }\left(\xi \right) & = & \rho_{0}\left(\xi \right)={\left|C\right|}^{2}\int_{A_{0}}^{B_{0}}dx\;x^{3}\;{\left(\cosh\xi \right)}^{1-\sqrt{1+\frac{4\mu^{2}\omega^{2}x^{4}}{\hbar^{2}}}} \end{array}$$

(118) $$\begin{array}{ccc} & = & \frac{\hbar^{2}}{2\mu^{2}\omega^{2}}\frac{{\left|C\right|}^{2}}{4}\frac{\cosh\xi -\left(1+\ln\cosh^{z}\xi \right)\cosh^{1-z}\xi }{{\left(\ln\cosh\xi \right)}^{2}}{|}_{z=\sqrt{1+\frac{4\mu^{2}\omega^{2}A_{0}^{4}}{\hbar^{2}}}}^{\sqrt{1+\frac{4\mu^{2}\omega^{2}B_{0}^{4}}{\hbar^{2}}}}, \end{array}$$

(119) $$\begin{array}{ccc} \rho_{\tau }\left(0\right) & = & \frac{{\left|C\right|}^{2}}{4}\left(B_{0}^{4}-A_{0}^{4}\right), \end{array}$$

where $f\left(z\right){|}_{z=a}^{b}=f\left(b\right)-f\left(a\right)$. In order to compare (118) with (110), without invoking a cumbersome explicit formula for ${\left|C\right|}^{2}$, let us take $A_{0}=0$ and express (110) in terms of its value at ξ=0,

(120) $$\begin{array}{ccc} \rho_{\tau }\left(0\right) & = & \frac{3B_{0}}{2\sqrt{\pi }}{\left(\frac{\hbar }{\mu \omega }\right)}^{-1/2}=R\varrho_{\tau }\left(0\right), \end{array}$$

(121) $$\begin{array}{ccc} \varrho_{\tau }\left(r\right) & = & \frac{2\hbar^{2}}{{\tilde{\mu }}^{2}\omega^{2}}\varrho_{\tau }\left(0\right)\frac{1-e^{-\frac{\tilde{\mu }\omega }{\hbar }r^{2}}\left(\frac{\tilde{\mu }\omega }{\hbar }r^{2}+1\right)}{r^{4}}, \end{array}$$

where $\tilde{\mu }=\mu B_{0}^{2}/R^{2}$. Analogously, setting $A_{0}=0$ in (118), we find

(122) $$\begin{array}{ccc} \varrho_{\tau }\left(r\right) & = & \frac{2\hbar^{2}}{{\tilde{\mu }}^{2}\omega^{2}}\varrho_{\tau }\left(0\right)\frac{\left(1+\ln\cosh^{z}\left(r/R\right)\right)\cosh^{1-z}\left(r/R\right)}{4R^{4}{\left(\ln\cosh\left(r/R\right)\right)}^{2}}{|}_{z=\sqrt{1+\frac{4{\tilde{\mu }}^{2}\omega^{2}R^{4}}{\hbar^{2}}}}^{1}. \end{array}$$

Now, one can directly verify that (121) is the R→∞ limit of (122). The limit is taken with $\tilde{\mu }=\mathrm{const}$.

## 8. A Two-Body Problem

A single harmonic oscillator in space is, in its simplest version, an example of a two-body problem with the potential $V(x_{1}-x_{2})$ that depends on the relative coordinate of the two bodies; the center-of-mass coordinate is typically subject to a free motion. An analogous situation is encountered in the problem we discuss in the present paper. So far, we have concentrated on the analysis of the relative coordinate, but one should be able to extend the analysis to the entire configuration space, involving an arbitrary number of particles. The two-body problem is thus the first step. We begin with

(123) $$\begin{array}{ccc} i{\dot{\psi }}_{\tau }\left(x_{1},\xi_{1},\theta_{1},\varphi_{1},x_{2},\xi_{2},\theta_{2},\varphi_{2}\right) & = & -i\ell^{4}\frac{\partial }{\partial (x_{1}^{4})}\psi_{\tau }\left(x_{1},\xi_{1},\theta_{1},\varphi_{1},x_{2},\xi_{2},\theta_{2},\varphi_{2}\right)\\ & & -i\ell^{4}\frac{\partial }{\partial (x_{2}^{4})}\psi_{\tau }\left(x_{1},\xi_{1},\theta_{1},\varphi_{1},x_{2},\xi_{2},\theta_{2},\varphi_{2}\right)\\ & & +\epsilon \left(-\frac{\hbar^{2}}{2m_{1}}\Delta_{x_{1}}-\frac{\hbar^{2}}{2m_{2}}\Delta_{x_{2}}+U\left(x_{1},\xi_{1},x_{2},\xi_{2}\right)\right)\psi_{\tau }\left(x_{1},\xi_{1},\theta_{1},\varphi_{1},x_{2},\xi_{2},\theta_{2},\varphi_{2}\right). \end{array}$$

Restricting our analysis to the l=0 cases, we can separate the angular variables and concentrate on

(124) $$\begin{array}{ccc} i{\dot{\phi }}_{\tau }\left(x_{1},\xi_{1},x_{2},\xi_{2}\right) & = & -i\ell^{4}\frac{\partial }{\partial (x_{1}^{4})}\phi_{\tau }\left(x_{1},\xi_{1},x_{2},\xi_{2}\right)-i\ell^{4}\frac{\partial }{\partial (x_{2}^{4})}\phi_{\tau }\left(x_{1},\xi_{1},x_{2},\xi_{2}\right)\\ & & +\epsilon \left(-\frac{\hbar^{2}}{2m_{1}}\Delta_{x_{1}}-\frac{\hbar^{2}}{2m_{2}}\Delta_{x_{2}}+U\left(x_{1},\xi_{1},x_{2},\xi_{2}\right)\right)\phi_{\tau }\left(x_{1},\xi_{1},x_{2},\xi_{2}\right), \end{array}$$

where

(125) $$\begin{array}{ccc} \Delta_{x_{k}} & = & \frac{1}{x_{k}^{2}\sinh^{2}\xi_{k}}\frac{\partial }{\partial \xi_{k}}\sinh^{2}\xi_{k}\frac{\partial }{\partial \xi_{k}},\;k=1,2. \end{array}$$

Switching to the interaction picture,

(126) $$\begin{array}{ccc} \Phi_{\tau }\left(x_{1},\xi_{1},x_{2},\xi_{2}\right) & = & e^{\tau \ell^{4}\left(\frac{\partial }{\partial (x_{1}^{4})}+\frac{\partial }{\partial (x_{2}^{4})}\right)}\phi_{\tau }\left(x_{1},\xi_{1},x_{2},\xi_{2}\right) \end{array}$$

(127) $$\begin{array}{ccc} & = & \phi_{\tau }\left(x_{1}\left(\tau \right),\xi_{1},x_{2}\left(\tau \right),\xi_{2}\right), \end{array}$$

(128) $$\begin{array}{ccc} x_{k}\left(\tau \right) & = & \sqrt[{4}]{x_{k}^{4}+\ell^{4}\tau }, \end{array}$$

we find

(129) $$\begin{array}{ccc} i{\dot{\Phi }}_{\tau }\left(x_{1},\xi_{1},x_{2},\xi_{2}\right) & = & \epsilon \left(-\frac{\hbar^{2}}{2m_{1}}\Delta_{x_{1}\left(\tau \right)}-\frac{\hbar^{2}}{2m_{2}}\Delta_{x_{2}\left(\tau \right)}+U\left(x_{1}\left(\tau \right),\xi_{1},x_{2}\left(\tau \right),\xi_{2}\right)\right)\Phi_{\tau }\left(x_{1},\xi_{1},x_{2},\xi_{2}\right). \end{array}$$

Now, let

(130) $$\begin{array}{c} F_{\tau }\left(x_{1},\xi_{1},x_{2},\xi_{2}\right)=\Phi_{\tau }\left(x_{1},\xi_{1},x_{2},\xi_{2}\right)\sinh\xi_{1}\sinh\xi_{2}. \end{array}$$

Employing $g^{-2}{\left(g^{2}{\left(f/g\right)}^{\prime }\right)}^{\prime }=g^{-1}\left(f^{\prime \prime }-fg^{\prime \prime }/g\right)$, we obtain

(131) $$\begin{array}{ccc} & & i{\dot{F}}_{\tau }\left(x_{1},\xi_{1},x_{2},\xi_{2}\right)\\ & & =\epsilon \left[-\frac{\hbar^{2}}{2m_{1}x_{1}{\left(\tau \right)}^{2}}\left(\frac{\partial^{2}}{\partial \xi_{1}^{2}}-1\right)-\frac{\hbar^{2}}{2m_{2}x_{2}{\left(\tau \right)}^{2}}\left(\frac{\partial^{2}}{\partial \xi_{2}^{2}}-1\right)+U\left(x_{1}\left(\tau \right),\xi_{1},x_{2}\left(\tau \right),\xi_{2}\right)\right]F_{\tau }\left(x_{1},\xi_{1},x_{2},\xi_{2}\right). \end{array}$$

An analogue of the center-of-mass system of coordinates is defined by

(132) $$\begin{array}{ccc} \Xi & = & \frac{m_{1}x_{1}{\left(\tau \right)}^{2}\xi_{1}+m_{2}x_{2}{\left(\tau \right)}^{2}\xi_{2}}{m_{1}x_{1}{\left(\tau \right)}^{2}+m_{2}x_{2}{\left(\tau \right)}^{2}}, \end{array}$$

(133) $$\begin{array}{ccc} \xi & = & \xi_{2}-\xi_{1}. \end{array}$$

Repeating standard calculations, we arrive at the interaction-picture Schrödinger equation,

(134) $$\begin{array}{ccc} & & i{\dot{G}}_{\tau }\left(x_{1},\Xi ,x_{2},\xi \right)\\ & & =\epsilon \left(-\frac{\hbar^{2}}{2I\left(x_{1}\left(\tau \right),x_{2}\left(\tau \right)\right)}\frac{\partial^{2}}{\partial \Xi^{2}}-\frac{\hbar^{2}}{2\iota \left(x_{1}\left(\tau \right),x_{2}\left(\tau \right)\right)}\frac{\partial^{2}}{\partial \xi^{2}}+V\left(x_{1}\left(\tau \right),\Xi ,x_{2}\left(\tau \right),\xi \right)\right)G_{\tau }\left(x_{1},\Xi ,x_{2},\xi \right), \end{array}$$

involving reduced and total moments of inertia (rather than reduced and total masses),

(135) $$\begin{array}{ccc} \frac{1}{\iota \left(x_{1},x_{2}\right)} & = & \frac{1}{m_{1}x_{1}^{2}}+\frac{1}{m_{2}x_{2}^{2}}, \end{array}$$

(136) $$\begin{array}{ccc} I\left(x_{1},x_{2}\right) & = & m_{1}x_{1}^{2}+m_{2}x_{2}^{2}, \end{array}$$

the wave function,

(137) $$\begin{array}{ccc} G_{\tau }\left(x_{1},\Xi ,x_{2},\xi \right) & = & F_{\tau }\left(x_{1},\Xi -\frac{m_{2}x_{2}^{2}}{m_{2}x_{2}^{2}+m_{1}x_{1}^{2}}\xi ,x_{2},\Xi +\frac{m_{1}x_{1}^{2}}{m_{2}x_{2}^{2}+m_{1}x_{1}^{2}}\xi \right), \end{array}$$

and the potential

(138) $$\begin{array}{ccc} V(x_{1},\Xi ,x_{2},\xi ) & = & \frac{\hbar^{2}}{2\iota (x_{1},x_{2})}+U\left(x_{1},\Xi -\frac{m_{2}x_{2}^{2}}{m_{2}x_{2}^{2}+m_{1}x_{1}^{2}}\xi ,x_{2},\Xi +\frac{m_{1}x_{1}^{2}}{m_{2}x_{2}^{2}+m_{1}x_{1}^{2}}\xi \right). \end{array}$$

In the limiting case $m_{1}\to \infty$, one reconstructs the formalism we have used so far, with

(139) $$\begin{array}{ccc} \xi_{1} & = & \Xi , \end{array}$$

(140) $$\begin{array}{ccc} \xi_{2} & = & \Xi +\xi , \end{array}$$

and

(141) $$\begin{array}{ccc} i{\dot{G}}_{\tau }\left(x_{1},\Xi ,x_{2},\Xi +\xi \right) & = & \epsilon \left(-\frac{\hbar^{2}}{2m_{2}x_{2}{\left(\tau \right)}^{2}}\frac{\partial^{2}}{\partial \xi^{2}}+V\left(x_{1}\left(\tau \right),\Xi ,x_{2}\left(\tau \right),\Xi +\xi \right)\right)G_{\tau }\left(x_{1},\Xi ,x_{2},\Xi +\xi \right). \end{array}$$

The harmonic oscillator example corresponds to Ξ=0, $m_{2}=\mu$, $x_{2}=x$, and

(142) $$\begin{array}{c} V\left(x_{1},0,x,\xi \right)=\frac{\mu \omega^{2}x^{2}\tanh^{2}\xi }{2}. \end{array}$$

For large values of τ, both $x_{1}$ and $x_{2}$ are localized in a neighborhood of $x=\ell \sqrt[{4}]{\tau }$.

## 9. Conclusions

The discussed formalism is meant as a unification and generalization of both standard cosmology and quantum mechanics. As opposed to classical cosmology, the universe is not represented by a spatial section of some space–time but by the support of a wave function propagating through space–time. Quantum mechanics known from textbooks are reconstructed asymptotically, for large times, by means of an appropriate correspondence principle. The universe is in general deformed by the presence of matter. We have decided to perform an explicit analysis of a simple but physically meaningful and exactly solvable system, hence, the choice of a harmonic oscillator. Among various possibilities, we have chosen the CRS model of the quantum harmonic oscillator, very natural in the context of spaces with constant curvature. Yet, as opposed to the original CRS formalism, the curvature in our formalism is not a parameter but a quantum observable. The resulting universe exists in a quantum superposition of different curvatures.

A general conclusion is that for late times, the evolution of the oscillator is essentially the one we know from standard quantum mechanics but with one important subtlety. Namely, the effective renormalized mass of the oscillator (inferred on the basis of the uncertainty of its geodesic distance r) is time-dependent, as opposed to the bare mass that occurs in the Hamiltonian. The time in $e^{-i\omega (n+1/2)t}$ is asymptotically (i.e., for late times) proportional to the quantum evolution parameter, t∼τ, the age of the universe is proportional to $\sqrt[{4}]{\tau }$, and the renormalized mass decays as $1/\sqrt{\tau }$. The effect does not seem to be a peculiarity of this concrete potential. Rather, it is a consequence of the concrete form of the empty-universe Hamiltonian and its coupling with matter. Since the renormalization of mass is influenced by the dynamics of the universe, the effect may be regarded as yet another version of Mach’s principle.

## Figures and Tables

**Figure A1:**
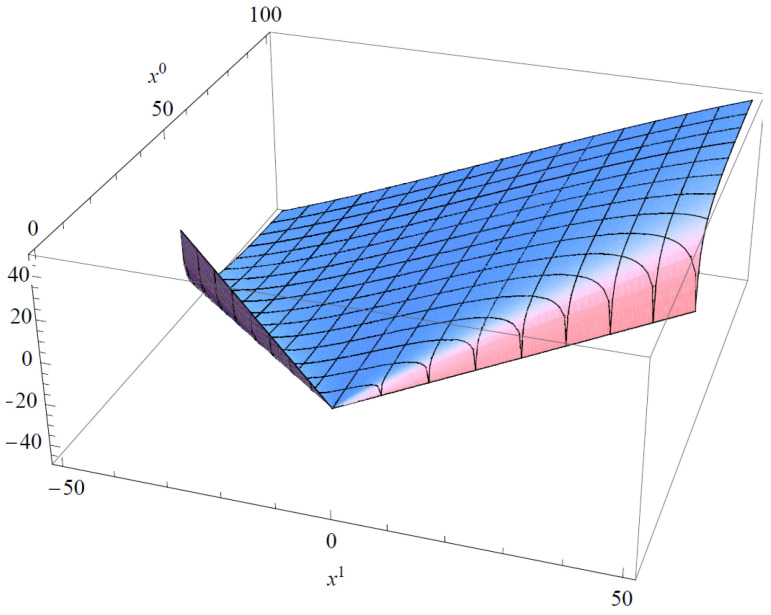
Geodesic coordinate $r(x^{0},x^{1})$ given by (A53) as a function of the Minkowski-space coordinates. $r(x^{0},x^{1})$ decays to 0 as *x* approaches the light cone x=0, a property that does not contradict the normalization by means of $\int_{-\infty }^{\infty }dr$, because the integral is computed along the hyperboloid x=const>0. Non-relativistic, late-time asymptotics of the position operator follow from the pointwise but non-uniform convergence $\lim_{x^{0}\to \infty }r\left(x^{0},x^{1}\right)=x^{1}$. The boundary condition, $\Psi_{\tau }\left(x\right)=0$ if x=0, implies that the light cone does not belong to the universe.

**Figure A2:**
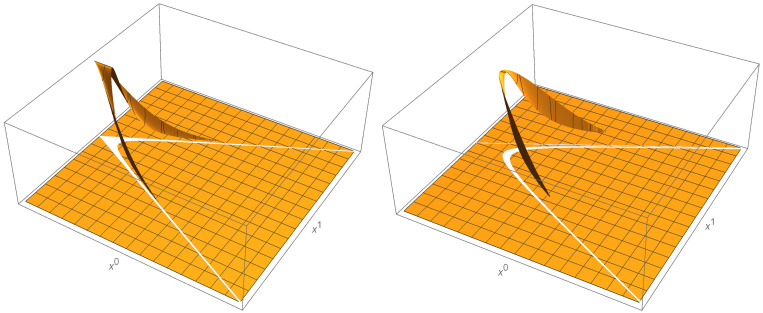
An analogue of Figure 2 but for the solution (A66). The parameters are $B_{0}=1$, mω/ℏ=1, ℓ=1, τ=0 (left), and τ=2 (right). The white region represents the support of the wave function, i.e., the configuration-space universe as a subset of the Minkowski space in D=1+1, characterized by $\Phi_{0,\tau }\left(x^{0},x^{1}\right)\neq 0$. The mass is given by its bare value.

## Data Availability

The original contributions presented in this study are included in the article. Further inquiries can be directed to the corresponding author.

## Appendix A

### Self-Adjointness, Unitarity, and Arrow of Time: Euclidean 2-Dimensional Case Study

In order to better understand the issues related to self-adjointness of the Hamiltonian and the problems of unitarity, let us simplify the discussion by considering an analogous problem but in the two dimensional plane $R^{2}$. The resulting example may be treated as a toy model of a closed positive-curvature quantum universe—the universe identified with some rotationally invariant layer in two Euclidean dimensions, a support of some wave function.

Consider the Hilbert space of square-integrable functions $f:R^{2}\to C$, with the scalar product

(A1) $$\begin{array}{ccc} \langle f|g\rangle & = & \int_{0}^{\infty }dr\;r\int_{0}^{2\pi }d\varphi \;\bar{f(r,\varphi )}g\left(r,\varphi \right), \end{array}$$

and the Schrödinger equation analogous to (3),

(A2) $$\begin{array}{ccc} i\frac{d\psi_{\tau }\left(r,\varphi \right)}{d\tau } & = & H\psi_{\tau }\left(r,\varphi \right)=-i\frac{\ell^{D}}{Dr^{D}}r\frac{\partial \psi_{\tau }\left(r,\varphi \right)}{\partial r} \end{array}$$

(A3) $$\begin{array}{ccc} & = & -i\frac{\ell^{2}}{2r}\frac{\partial \psi_{\tau }\left(r,\varphi \right)}{\partial r}=-i\ell^{2}\frac{\partial \psi_{\tau }\left(r,\varphi \right)}{\partial (r^{2})}, \end{array}$$

where D=2 is the dimension of the Euclidean space $R^{2}$. Definition (A2) is analogous to (3), but instead of the background (1+3)-dimensional Minkowski space, we consider the ordinary Euclidean plane. The coordinates are the usual polar *r* and φ. Let us now change variables, $\rho =r^{2}$, and define the new function

(A4) $$\begin{array}{c} F_{\tau }\left(\rho ,\varphi \right)=\psi_{\tau }\left(\sqrt{\rho },\varphi \right), \end{array}$$

so that the Schrödinger equation, as well as its general solution, reads

(A5) $$\begin{array}{ccc} \frac{dF_{\tau }\left(\rho ,\varphi \right)}{d\tau } & = & -\ell^{2}\frac{\partial F_{\tau }\left(\rho ,\varphi \right)}{\partial \rho }, \end{array}$$

(A6) $$\begin{array}{ccc} F_{\tau }\left(\rho ,\varphi \right) & = & e^{-\tau \ell^{2}\frac{\partial }{\partial \rho }}F_{0}\left(\rho ,\varphi \right)=F_{0}\left(\rho -\tau \ell^{2},\varphi \right)=F_{0}\left(\rho_{\tau },\varphi \right)=\psi_{0}\left(\sqrt{\rho_{\tau }},\varphi \right) \end{array}$$

(A7) $$\begin{array}{ccc} & = & \psi_{0}\left(\sqrt{r^{2}-\tau \ell^{2}},\varphi \right)=\psi_{\tau }\left(r,\varphi \right). \end{array}$$

Formally, (A5) is the wave equation describing waves propagating to the right with velocity $\ell^{2}$. As the initial condition, we will take a function that is non-zero only in the open ring $r_{1}<r<r_{2}$, with some non negative $r_{1},r_{2}$. Assume for simplicity,

(A8) $$\begin{array}{c} \psi_{0}\left(r,\varphi \right)=\frac{1}{\sqrt{\pi (r_{2}^{2}-r_{1}^{2})}}\chi_{]r_{1},r_{2}[}\left(r\right), \end{array}$$

where $\chi_{]r_{1},r_{2}[}\left(r\right)$ is a smooth compact-support function of the type discussed in Figure 1, approximating with arbitrary accuracy the characteristic function of the open interval $]r_{1},r_{2}[$. The initial condition is normalized,

(A9) $$\begin{array}{ccc} \langle \psi_{0}|\psi_{0}\rangle & = & \int_{0}^{\infty }dr\;r\int_{0}^{2\pi }d\varphi \;{\left|\psi_{0}\left(r,\varphi \right)\right|}^{2} \end{array}$$

(A10) $$\begin{array}{ccc} & = & \frac{1}{\pi (r_{2}^{2}-r_{1}^{2})}\int_{r_{1}}^{r_{2}}dr\;r\int_{0}^{2\pi }d\varphi =1. \end{array}$$

The form (A7) of the solution implies that $\psi_{\tau }\left(r,\varphi \right)$ is non-vanishing only for

(A11) $$\begin{array}{c} r_{1}<\sqrt{r^{2}-\tau \ell^{2}}<r_{2}. \end{array}$$

which is equivalent to

(A12) $$\begin{array}{c} \sqrt{r_{1}^{2}+\tau \ell^{2}}<r<\sqrt{r_{2}^{2}+\tau \ell^{2}}. \end{array}$$

Accordingly,

(A13) $$\begin{array}{ccc} \langle \psi_{\tau }|\psi_{\tau }\rangle & = & \int_{0}^{\infty }dr\;r\int_{0}^{2\pi }d\varphi \;{\left|\psi_{\tau }\left(r,\varphi \right)\right|}^{2} \end{array}$$

(A14) $$\begin{array}{ccc} & = & \frac{1}{\pi (r_{2}^{2}-r_{1}^{2})}\int_{\sqrt{r_{1}^{2}+\tau \ell^{2}}}^{\sqrt{r_{2}^{2}+\tau \ell^{2}}}dr\;r\int_{0}^{2\pi }d\varphi =1. \end{array}$$

Let us note that (A12) is well defined for the evolution parameter τ, restricted by

(A15) $$\begin{array}{c} -r_{1}^{2}/\ell^{2}\leq \tau , \end{array}$$

which means that τ in both

(A16) $$\begin{array}{ccc} F_{\tau }\left(\rho ,\varphi \right) & = & e^{-\tau \ell^{2}\frac{\partial }{\partial \rho }}F_{0}\left(\rho ,\varphi \right) \end{array}$$

and

(A17) $$\begin{array}{c} \langle \psi_{\tau }|\psi_{\tau }\rangle =1 \end{array}$$

is restricted by (A15) as well. The geometric interpretation of this property is obvious: For negative τ, the evolution given by (A16) shifts $F_{\tau }\left(\rho ,\varphi \right)$ to the left along the ρ coordinate, but $\rho =r^{2}$ cannot become negative on $R^{2}$. Moreover, by the same inequality (A15), we observe that the possible range of available τ depends on the form of the initial condition. The interpretation of the latter property is also evident: An available time of propagation to the left depends on how far to the right we begin.

For $\tau <-r_{1}^{2}/\ell^{2}$, we obtain

(A18) $$\begin{array}{c} \langle \psi_{\tau }|H\psi_{\tau }\rangle \neq \langle H\psi_{\tau }|\psi_{\tau }\rangle , \end{array}$$

because the boundary terms arising from integration by parts will no longer cancel each other. All these properties are typical of a *unitary semigroup*, possessing an arrow of time, and this is why H is not self-adjoint. Any attempt of rescuing self-adjointness by continuing $r^{2}$ to negative values would bring us outside of the real plane, contradicting our assumption that the waves propagate on $R^{2}$.

On the other hand, for τ satisfying (A15), the norm of the solution equals 1, so this is just a wave that represents some probability density radially propagating on $R^{2}$. As opposed to standard circular waves created by a stone thrown into a lake, the resulting wave does not decrease its height, and does not spread along the radial direction. Just the opposite, this is like a solitary wave whose radial width shrinks to zero so that the mass contained in the volume of the wave remains unchanged.

There is no problem with analyzing this type of wave propagation by means of Fourier analysis, with the Fourier modes playing the role of the basis in the Hilbert space in question. Interestingly, the fact that the support of $\psi_{\tau }$ is a ring of finite radius and width implies that the resulting Fourier modes will be discrete for any τ. The Fourier analysis effectively replaces the missing spectral theory of the Hamiltonian. We do not make any attempt of formulating a theory of measurement, as the universe that contains only a single harmonic oscillator involves no laboratory observers.

As a final remark, let us note that the presence of $r^{D}$ and $x^{D}$ in the denominators of (A2) and (3) distinguishes these Hamiltonians from dilatation generators r∂/∂r and $x^{\mu }\partial_{\mu }$. Our Hamiltonians do not generate dilatations of *r* or x but *translations* of $r^{D}$ and $x^{D}$, which turns out to be equivalent to a form of squeezing.

## Appendix B

### Appendix B.1. Excited States for sinhξ≈tanhξ≈ξ

So far, we have concentrated on the ground states, but it is a simple exercise to derive all the excited states as long as we work in the approximate model, valid for sinhξ≈tanhξ≈ξ. All the excited states should lead to coherent states and a semiclassical limit of our theory, the problem worthy of a separate study. The exact CRS model is much more complicated but still exactly solvable by a combination of the results from [14,15] with what we describe in the present paper. It must be kept in mind that potentials proportional to $\tanh^{2}\xi$ are bounded from above, and thus, only a finite number of discrete-energy states are expected to occur. The same subtlety is found in the Kepler problem, as discussed in [24].

For simplicity, let us consider D=1+1 so that the angular coordinates are absent from the very outset. The main difference between D=1+1 and D=1+3, l=0, is in the form of the free Hamiltonian, a generator of translations of $x^{D}$. In case of a Gaussian, the variable $x^{2}$ is replaced in interaction picture by $x^{2}+\ell^{2}\tau$, for D=1+1, and by $\sqrt{x^{4}+\ell^{4}\tau }$, for D=1+3. In various other respects the two cases are qualitatively similar.

Assume

(A19) $$\begin{array}{ccc} U(x_{1},x,\xi ) & = & \frac{\iota \left(x\right)\omega^{2}\xi^{2}}{2}, \end{array}$$

where ι(x) is a moment of inertia to be specified later. Switching to the interaction picture,

(A20) $$\begin{array}{ccc} {\tilde{\phi }}_{\tau }\left(x_{1},\xi_{1},x,\xi \right) & = & e^{\tau \ell^{2}\left(\frac{\partial }{\partial (x_{1}^{2})}+\frac{\partial }{\partial (x^{2})}\right)}\phi_{\tau }\left(x_{1},\xi_{1},x,\xi \right) \end{array}$$

(A21) $$\begin{array}{ccc} & = & \phi_{\tau }\left(\sqrt{x_{1}^{2}+\tau \ell^{2}},\xi_{1},\sqrt{x^{2}+\tau \ell^{2}},\xi \right), \end{array}$$

we obtain a τ-dependent Hamiltonian,

(A22) $$\begin{array}{ccc} i\frac{d}{d\tau }{\tilde{\phi }}_{\tau }\left(x_{1},\xi_{1},x,\xi \right) & = & \epsilon \left(-\frac{\hbar^{2}}{2\iota \left(\sqrt{x^{2}+\tau \ell^{2}}\right)}\frac{\partial^{2}}{\partial \xi^{2}}+\frac{\iota \left(\sqrt{x^{2}+\tau \ell^{2}}\right)\omega^{2}\xi^{2}}{2}\right){\tilde{\phi }}_{\tau }\left(x_{1},\xi_{1},x,\xi \right) \end{array}$$

(A23) $$\begin{array}{ccc} & = & \epsilon \hbar \omega \left(a{\left(\sqrt{x^{2}+\tau \ell^{2}}\right)}^{†}a\left(\sqrt{x^{2}+\tau \ell^{2}}\right)+\frac{1}{2}\right){\tilde{\phi }}_{\tau }\left(x_{1},\xi_{1},x,\xi \right), \end{array}$$

where

(A24) $$\begin{array}{ccc} a(x) & = & \frac{1}{\sqrt{\hbar \omega }}\left(\frac{\hbar }{\sqrt{2\iota (x)}}\frac{\partial }{\partial \xi }+\sqrt{\frac{\iota (x)}{2}}\omega \xi \right), \end{array}$$

(A25) $$\begin{array}{ccc} a{\left(x\right)}^{†} & = & \frac{1}{\sqrt{\hbar \omega }}\left(-\frac{\hbar }{\sqrt{2\iota (x)}}\frac{\partial }{\partial \xi }+\sqrt{\frac{\iota (x)}{2}}\omega \xi \right). \end{array}$$

The ground state

(A26) $$\begin{array}{ccc} {\tilde{\phi }}_{0,\tau }\left(x_{1},\xi_{1},x,\xi \right) & = & \phi_{0,\tau }\left(\sqrt{x_{1}^{2}+\tau \ell^{2}},\xi_{1},\sqrt{x^{2}+\tau \ell^{2}},\xi \right), \end{array}$$

is defined by

(A27) $$\begin{array}{ccc} a\left(\sqrt{x^{2}+\tau \ell^{2}}\right){\tilde{\phi }}_{0,\tau }\left(x_{1},\xi_{1},x,\xi \right) & = & a\left(\sqrt{x^{2}+\tau \ell^{2}}\right)\phi_{0,\tau }\left(\sqrt{x_{1}^{2}+\tau \ell^{2}},\xi_{1},\sqrt{x^{2}+\tau \ell^{2}},\xi \right)=0. \end{array}$$

Following the standard steps one proves

(A28) $$\begin{array}{ccc} i\frac{d}{d\tau }{\tilde{\phi }}_{n,\tau }\left(x_{1},\xi_{1},x,\xi \right) & = & \epsilon \hbar \omega \left(n+\frac{1}{2}\right){\tilde{\phi }}_{n,\tau }\left(x_{1},\xi_{1},x,\xi \right), \end{array}$$

(A29) $$\begin{array}{ccc} {\tilde{\phi }}_{n,\tau }\left(x_{1},\xi_{1},x,\xi \right) & = & e^{-i\epsilon \hbar \omega \left(n+\frac{1}{2}\right)\tau }{\tilde{\phi }}_{n,0}\left(x_{1},\xi_{1},x,\xi \right), \end{array}$$

or equivalently,

(A30) $$\begin{array}{c} \phi_{n,\tau }\left(\sqrt{x_{1}^{2}+\tau \ell^{2}},\xi_{1},\sqrt{x^{2}+\tau \ell^{2}},\xi \right)=e^{-i\epsilon \hbar \omega \left(n+\frac{1}{2}\right)\tau }\phi_{n,0}\left(x_{1},\xi_{1},x,\xi \right) \end{array}$$

and thus,

(A31) $$\begin{array}{c} \phi_{n,\tau }\left(x_{1},\xi_{1},x,\xi \right)=e^{-i\epsilon \hbar \omega \left(n+\frac{1}{2}\right)\tau }\phi_{n,0}\left(\sqrt{x_{1}^{2}-\tau \ell^{2}},\xi_{1},\sqrt{x^{2}-\tau \ell^{2}},\xi \right), \end{array}$$

with

(A32) $$\begin{array}{ccc} \phi_{n,0}\left(x_{1},\xi_{1},x,\xi \right) & = & \frac{1}{\sqrt{2^{n}n!}}f\left(x_{1},\xi_{1},x\right)H_{n}\left(\sqrt{\frac{\iota (x)\omega }{\hbar }}\xi \right)e^{-\frac{\iota (x)\omega }{2\hbar }\xi^{2}}, \end{array}$$

(A33) $$\begin{array}{ccc} H_{n}\left(\eta \right) & = & {\left(-1\right)}^{n}e^{\eta^{2}}\frac{d^{n}}{d\eta^{n}}e^{-\eta^{2}}, \end{array}$$

and $f\left(x_{1},\xi_{1},x\right)$ still unspecified. Formula (A31) shows that the usual quantum mechanical time parameter can be identified with t=ϵℏτ. Note that t is as invariant as τ and x and thus cannot be regarded as a timelike coordinate $t=x^{0}/c$ of a world-position.

Let us recall that in $m_{1}\to \infty$ limit. the first two coordinates,

(A34) $$\begin{array}{ccc} x_{1} & = & \left(x_{1}^{0},x_{1}^{1}\right)=x_{1}\left(\cosh\xi_{1},\sinh\xi_{1}\right), \end{array}$$

describe the center-of-mass spacetime-position operator of the system. We can consider an initial condition that separates the center of mass coordinate $x_{1}^{\mu }$ from $x^{\mu }$, the relative one, and concentrate on the latter,

(A35) $$\begin{array}{ccc} \phi_{n,\tau }\left(x,\xi \right) & = & e^{-i\epsilon \hbar \omega \left(n+\frac{1}{2}\right)\tau }\frac{1}{\sqrt{2^{n}n!}}f\left(\sqrt{x^{2}-\tau \ell^{2}}\right)H_{n}\left(\sqrt{\frac{\omega \iota \left(\sqrt{x^{2}-\tau \ell^{2}}\right)}{\hbar }}\xi \right)e^{-\frac{\omega }{2\hbar }\iota \left(\sqrt{x^{2}-\tau \ell^{2}}\right)\xi^{2}}. \end{array}$$

It is subject to normalization

(A36) $$\begin{array}{ccc} 1 & = & \langle \phi_{n,\tau }|\phi_{n,\tau }\rangle =\langle \phi_{n,0}|\phi_{n,0}\rangle =\langle \phi_{0,0}|\phi_{0,0}\rangle \end{array}$$

(A37) $$\begin{array}{ccc} & = & \int_{0}^{\infty }dx\;x{\left|f\left(x\right)\right|}^{2}\int_{-\infty }^{\infty }d\xi e^{-\frac{\omega }{\hbar }\iota \left(x\right)\xi^{2}} \end{array}$$

(A38) $$\begin{array}{ccc} & = & \sqrt{\frac{\pi \hbar }{\omega }}\int_{0}^{\infty }dx\frac{{x|f\left(x\right)|}^{2}}{\sqrt{\iota (x)}}. \end{array}$$

In (A37), we integrate over ξ∈R and not over $\xi \in R_{+}$, a peculiarity of the 1+1 case.

#### Appendix B.1.1. Constant Moment of Inertia, ι(x)=mR 2

For $\iota \left(x\right)=mR^{2}$ (i.e., in a flat universe), we obtain

(A39) $$\begin{array}{ccc} \phi_{n,\tau }\left(x,\xi \right) & = & e^{-i\epsilon \hbar \omega \left(n+\frac{1}{2}\right)\tau }\frac{1}{\sqrt{2^{n}n!}}f\left(\sqrt{x^{2}-\tau \ell^{2}}\right)H_{n}\left(\sqrt{\frac{m\omega }{\hbar }}R\xi \right)e^{-\frac{m\omega }{2\hbar }R^{2}\xi^{2}}, \end{array}$$

with

(A40) $$\begin{array}{c} A_{0}^{2}<x^{2}-\ell^{2}\tau <B_{0}^{2}. \end{array}$$

This is, essentially, an oscillator described in terms of some geodesic coordinate r=Rξ and time t=ϵℏτ.

#### Appendix B.1.2. x-Dependent Moment of Inertia, ι(x)=mx 2

For $\iota \left(x\right)=mx^{2}$ (i.e., in the hyperbolic universe), the non-vanishing part of the solution reads

(A41) $$\begin{array}{ccc} \phi_{n,\tau }\left(x,\xi \right) & = & e^{-i\epsilon \hbar \omega \left(n+\frac{1}{2}\right)\tau }\frac{1}{\sqrt{2^{n}n!}}f\left(\sqrt{x^{2}-\ell^{2}\tau }\right)H_{n}\left(\sqrt{\frac{m\omega }{\hbar }}\sqrt{x^{2}-\ell^{2}\tau }\xi \right)e^{-\frac{m\omega }{2\hbar }\left(x^{2}-\ell^{2}\tau \right)\xi^{2}}, \end{array}$$

with

(A42) $$\begin{array}{c} A_{0}^{2}<x^{2}-\ell^{2}\tau <B_{0}^{2}. \end{array}$$

#### Appendix B.1.3. Renormalization of Mass

Define r=xξ. For $A_{0}^{2}<x^{2}-\ell^{2}\tau <B_{0}^{2}$, rewrite (A35) as

(A43) $$\begin{array}{ccc} {\overset{ˇ}{\phi }}_{n,t}\left(x,r\right) & = & e^{-i\omega \left(n+\frac{1}{2}\right)t}\frac{1}{\sqrt{2^{n}n!}}f\left(\sqrt{x^{2}-\ell^{2}\tau }\right)H_{n}\left(\sqrt{\frac{m\omega }{\hbar }}\gamma \left(t,x\right)r\right)e^{-\frac{m\omega }{2\hbar }\gamma {\left(t,x\right)}^{2}r^{2}}, \end{array}$$

(A44) $$\begin{array}{ccc} \gamma (t,x) & = & \sqrt{\frac{\iota (\sqrt{x^{2}-\ell ct})}{mx^{2}}}=\left\{\begin{array}{cc} R/x & \mathrm{for}\iota ={\mathrm{mR}}^{2}\\ \sqrt{1-\ell^{2}\tau /x^{2}} & \mathrm{for}\iota =mx^{2} \end{array}\right., \end{array}$$

where

(A45) $$\begin{array}{c} A_{0}/x<\sqrt{1-\ell^{2}\tau /x^{2}}<B_{0}/x. \end{array}$$

Otherwise ${\overset{ˇ}{\phi }}_{n,t}\left(x,r\right)=0$.

An observer who performs position measurements in terms of r will conclude that the role of the parameter $\frac{m\omega }{\hbar }$ is played by its rescaled version $\frac{m\omega }{\hbar }\gamma {\left(t,x\right)}^{2}$. Simultaneously, one does not observe any modification of frequency in the oscillating term $e^{-i\omega \left(n+\frac{1}{2}\right)t}$. Accordingly, the rescaling of mω/ℏ has to be caused by a renormalization of m/ℏ, the parameter that controls the classical limit of the theory. It seems most natural to associate the effect with the renormalization of mass. Therefore, defining

(A46) $$\begin{array}{ccc} m(t,x) & = & m\gamma {\left(t,x\right)}^{2}, \end{array}$$

we find that the observed mass m(t,x) decays asymptotically, for late times, as $1/x^{2}∼1/t$. For D=1+3, the dependence on t is different, but still, m(t,x) asymptotically tends to zero. Observers performing quantum measurements in position representation defined by r=xξ may conclude that the present mass of the oscillator is smaller than its earlier values.

Let us note that the change in variables from (x,ξ) to (x,r) entails a modification of the form of $H_{0}$. Denoting

(A47) $$\begin{array}{ccc} \psi (x,\xi ) & = & \Psi \left(x\cosh\xi ,x\sinh\xi \right)=\Psi \left(x^{0},x^{1}\right), \end{array}$$

(A48) $$\begin{array}{ccc} \overset{ˇ}{\psi }\left(x,r\right) & = & \Psi \left(x\cosh\frac{r}{x},x\sinh\frac{r}{x}\right), \end{array}$$

and employing the proportionality between $H_{0}$ and Euler’s homogeneity operator, we find

(A49) $$\begin{array}{ccc} H_{0}\psi \left(x,\xi \right) & = & -i\ell^{2}\frac{\partial }{\partial (x^{2})}\psi \left(x,\xi \right), \end{array}$$

(A50) $$\begin{array}{ccc} H_{0}\overset{ˇ}{\psi }\left(x,r\right) & = & -i\frac{\ell^{2}}{2x^{2}}\left(x\frac{\partial }{\partial x}+r\frac{\partial }{\partial r}\right)\overset{ˇ}{\psi }\left(x,r\right). \end{array}$$

The inverse formulas read

(A51) $$\begin{array}{ccc} x & = & \sqrt{{\left(x^{0}\right)}^{2}-{\left(x^{1}\right)}^{2}}, \end{array}$$

(A52) $$\begin{array}{ccc} \xi & = & \ln\sqrt{\frac{x^{0}+x^{1}}{x^{0}-x^{1}}}, \end{array}$$

(A53) $$\begin{array}{ccc} r & = & \sqrt{{\left(x^{0}\right)}^{2}-{\left(x^{1}\right)}^{2}}\ln\sqrt{\frac{x^{0}+x^{1}}{x^{0}-x^{1}}}, \end{array}$$

(A54) $$\begin{array}{ccc} \Psi (x^{0},x^{1}) & = & \psi \left(\sqrt{{\left(x^{0}\right)}^{2}-{\left(x^{1}\right)}^{2}},\ln\sqrt{\frac{x^{0}+x^{1}}{x^{0}-x^{1}}}\right), \end{array}$$

whereas the corresponding scalar products are related by

(A55) $$\begin{array}{ccc} \langle \Psi |\Phi \rangle & = & \int_{V_{+}}dx\;\bar{\Psi (x)}\Phi \left(x\right) \end{array}$$

(A56) $$\begin{array}{ccc} & = & \int_{0}^{\infty }dx\int_{-\infty }^{\infty }dr\;\bar{\overset{ˇ}{\psi }\left(x,r\right)}\overset{ˇ}{\phi }\left(x,r\right) \end{array}$$

(A57) $$\begin{array}{ccc} & = & \int_{0}^{\infty }dx\;x\int_{-\infty }^{\infty }d\xi \;\bar{\psi (x,\xi )}\phi \left(x,\xi \right). \end{array}$$

#### Appendix B.1.4. Example: Mass vs. Effective Renormalized Mass (Hyperbolic Case)

Consider (A35), with n=0, $\iota =mx^{2}$ (the hyperbolic case), $A_{0}=0$, $B_{0}>0$,

(A58) $$\begin{array}{ccc} \phi_{0,\tau }\left(x,\xi \right) & = & e^{-\frac{i}{2}\epsilon \hbar \omega \tau }f\left(\sqrt{x^{2}-\tau \ell^{2}}\right)e^{-\frac{\omega m}{2\hbar }\left(x^{2}-\tau \ell^{2}\right)\xi^{2}} \end{array}$$

(A59) $$\begin{array}{ccc} & \approx & Ce^{-\frac{i}{2}\omega t}e^{-\frac{\omega }{2\hbar }m\left(1-\tau \ell^{2}/x^{2}\right)r^{2}},\;\mathrm{for}0<x^{2}-\tau \ell^{2}<B_{0}^{2} \end{array}$$

and 0 otherwise, normalized by (A38),

(A60) $$\begin{array}{ccc} 1 & = & \sqrt{\frac{\pi \hbar }{m\omega }}\int_{0}^{\infty }{dx|f\left(x\right)|}^{2}\approx \sqrt{\frac{\pi \hbar }{m\omega }}B_{0}{\left|C\right|}^{2}. \end{array}$$

The renormalized mass,

(A61) $$\begin{array}{c} \tilde{m}=m\left(1-\tau \ell^{2}/x^{2}\right), \end{array}$$

is the effective mass as measured by the width of the Gaussian, hence, by the uncertainty of the geodesic coordinate r=xξ. Now assume that $\tau_{0}$ is the current age of the universe, and thus,

(A62) $$\begin{array}{c} 0<{\tilde{m}}_{0}=m\left(1-\tau_{0}\ell^{2}/x^{2}\right)<B_{0}^{2}/x^{2}, \end{array}$$

is the current renormalized mass of, say, an electron oscillating with frequency ω. Note that x belongs to the support of the wave function at the current value of $\tau_{0}$. Denoting $t_{0}=\epsilon \hbar \tau_{0}$, $t_{0}+\delta t=\epsilon \hbar \tau_{0}+\epsilon \hbar \delta \tau$, we find

(A63) $$\begin{array}{ccc} \phi_{0,\tau_{0}+\delta \tau }\left(x,\xi \right) & \approx & Ce^{-\frac{i}{2}\omega \left(t_{0}+\delta t\right)}e^{-\frac{\omega }{2\hbar }m\left(1-\left(\tau_{0}+\delta \tau \right)\ell^{2}/x^{2}\right)r^{2}} \end{array}$$

(A64) $$\begin{array}{ccc} & \approx & Ce^{-\frac{i}{2}\omega \left(t_{0}+\delta t\right)}e^{-\frac{\omega }{2\hbar }{\tilde{m}}_{0}r^{2}}, \end{array}$$

if $\delta t/t_{0}\ll 1$, which can be safely assumed for all quantum mechanical experiments performed during the past century. Equation (A64) is the standard nonrelativistic result. However, if $\delta \tau \approx -\tau_{0}$, that is, we look at the state of the oscillator in a distant past, hence, for small x, the renormalized mass tends towards the bare mass, $\tilde{m}\approx m$, which is much larger.

The situation changes if we work with the bare mass. When expressed in space–time coordinates, $\left(x^{0},x^{1}\right)$, the solution evolves in configuration space–time as a squeezed state:

(A65) $$\begin{array}{ccc} \Phi_{0,\tau }\left(x^{0},x^{1}\right) & = & e^{-\frac{i}{2}\omega t}f\left(\sqrt{{\left(x^{0}\right)}^{2}-{\left(x^{1}\right)}^{2}-\tau \ell^{2}}\right)e^{-\frac{\omega m}{2\hbar }\left({\left(x^{0}\right)}^{2}-{\left(x^{1}\right)}^{2}-\tau \ell^{2}\right)\ln^{2}\sqrt{\frac{x^{0}+x^{1}}{x^{0}-x^{1}}}} \end{array}$$

(A66) $$\begin{array}{ccc} & \approx & Ce^{-\frac{i}{2}\omega t}e^{-\frac{\omega m}{2\hbar }\left({\left(x^{0}\right)}^{2}-{\left(x^{1}\right)}^{2}-\tau \ell^{2}\right)\ln^{2}\sqrt{\frac{x^{0}+x^{1}}{x^{0}-x^{1}}}}, \end{array}$$

if $0<{\left(x^{0}\right)}^{2}-{\left(x^{1}\right)}^{2}-\tau \ell^{2}<B_{0}^{2}$ and vanishes otherwise. Figure A2 illustrates the probability density associated with (A66) for some arbitrarily chosen dimensionless parameters.

## Appendix C

### An Alternative Definition of the Harmonic Oscillator

In the symmetry scattering approach to Schrödinger equations [25], a potential is identified with the angular part of an appropriate Laplace–Beltrami operator, hence, in our case, this would be the term proportional to l(l+1). Of course, this is not what we are interested in if we want to define a harmonic oscillator. Let us therefore try an alternative but equally general formulation.

We start with the observation that in nonrelativistic quantum mechanics, we find

(A67) $$\begin{array}{ccc} \Delta U(x) & = & \Delta \frac{m\omega^{2}x^{2}}{2}=3m\omega^{2}=\mathrm{const}>0. \end{array}$$

An analogous generalization can be formulated for any Laplace–Beltrami operator, in particular,

(A68) $$\begin{array}{ccc} & & \Delta_{x}U\left(x,\xi ,\theta ,\varphi \right)\\ & & =\frac{1}{x^{2}\sinh^{2}\xi }\frac{\partial }{\partial \xi }\sinh^{2}\xi \frac{\partial }{\partial \xi }U\left(x,\xi ,\theta ,\varphi \right)+\frac{1}{x^{2}\sinh^{2}\xi }\left(\frac{1}{\sin\theta }\frac{\partial }{\partial \theta }\sin\theta \frac{\partial }{\partial \theta }+\frac{1}{\sin^{2}\theta }\frac{\partial^{2}}{\partial \varphi^{2}}\right)U\left(x,\xi ,\theta ,\varphi \right)\\ & & =\mathrm{const}>0. \end{array}$$

So, first of all,

(A69) $$\begin{array}{c} U\left(x,\xi ,\theta ,\varphi \right)=x^{2}V\left(\xi ,\theta ,\varphi \right), \end{array}$$

and

(A70) $$\begin{array}{c} \frac{1}{\sinh^{2}\xi }\frac{\partial }{\partial \xi }\sinh^{2}\xi \frac{\partial }{\partial \xi }V\left(\xi ,\theta ,\varphi \right)+\frac{1}{\sinh^{2}\xi }\left(\frac{1}{\sin\theta }\frac{\partial }{\partial \theta }\sin\theta \frac{\partial }{\partial \theta }+\frac{1}{\sin^{2}\theta }\frac{\partial^{2}}{\partial \varphi^{2}}\right)V\left(\xi ,\theta ,\varphi \right)=C>0. \end{array}$$

Assuming rotational invariance, we obtain

(A71) $$\begin{array}{c} \frac{1}{\sinh^{2}\xi }\frac{\partial }{\partial \xi }\sinh^{2}\xi \frac{\partial }{\partial \xi }V\left(\xi \right)=C, \end{array}$$

whose general solution reads

(A72) $$\begin{array}{ccc} V(\xi ) & = & \frac{C}{2}\xi \coth\xi +C_{1}\coth\xi +C_{2}. \end{array}$$

For C=0, we reconstruct the Infeld–Schild version of the Coulomb–Newton potential [24]. It is intriguing that the oscillator and the Kepler problem are so intimately related, even at such a general level.

A finite value at ξ=0 implies $C_{1}=0$, and then

(A73) $$\begin{array}{ccc} \lim_{\xi \to 0}V\left(\xi \right) & = & \frac{C}{2}+C_{2}. \end{array}$$

We assume $C_{2}=-C/2$, so that V(0)=0. Then,

(A74) $$\begin{array}{ccc} U(x,\xi ,\theta ,\phi ) & = & \frac{C}{2}x^{2}\left(\xi \coth\xi -1\right)=\frac{C}{2}\frac{x^{2}\xi^{2}}{3}+\ldots \end{array}$$

The correspondence principle with standard quantum mechanics implies

(A75) $$\begin{array}{ccc} H & = & -\frac{\hbar^{2}}{2m}\Delta_{x}+\frac{3}{2}m\omega^{2}x^{2}\left(\xi \coth\xi -1\right) \end{array}$$

(A76) $$\begin{array}{ccc} & = & -\frac{\hbar^{2}}{2m}\Delta_{x}+\frac{m\omega^{2}r^{2}}{2}+\ldots \end{array}$$

where r=xξ is the geodesic coordinate, i.e.,

(A77) $$\begin{array}{ccc} \Delta_{x}U\left(x,\xi ,\theta ,\varphi \right) & = & 3m\omega^{2}, \end{array}$$

which is the same definition as in nonrelativistic quantum mechanics. For large ξ, the potential is linear,

(A78) $$\begin{array}{c} U\left(x,\xi ,\theta ,\varphi \right)=\frac{3}{2}m\omega^{2}x^{2}\left(\xi \coth\xi -1\right)\approx \frac{3}{2}m\omega^{2}x^{2}\left(\xi -1\right)=\frac{3}{2}m\omega^{2}x\left(r-x\right). \end{array}$$

As opposed to the CRS potential, Equation (A75) is expected to have infinitely many bound states and purely discrete spectrum (cf. [26]). Unfortunately, we have not managed to factorize (A75).
